# Estimating the burden of leptospirosis in the Caribbean: Insights from environmental and sociodemographic factors

**DOI:** 10.1371/journal.pntd.0013876

**Published:** 2026-07-06

**Authors:** Beatris Mario Martin, Zhonghan Zhang, Holly Jian, Sebastian Vernal, Eric J. Nilles, Luis Furuya-Kanamori, Benn Sartorius, Colleen L. Lau

**Affiliations:** 1 Frazer Institute, Faculty of Health, Medicine, and Behavioural Sciences, The University of Queensland, Brisbane, Australia; 2 National Centre for Epidemiology and Population Health, Australian National University, Acton, Australia; 3 Harvard Humanitarian Initiative, Cambridge, Massachusetts, United States of America; 4 Harvard Medical School, Boston, Massachusetts, United States of America; 5 Brigham and Women’s Hospital, Boston, Massachusetts, United States of America; 6 Department of Health Metric Sciences, University of Washington, Seattle, United States of America; London School of Hygiene & Tropical Medicine, UNITED KINGDOM OF GREAT BRITAIN AND NORTHERN IRELAND

## Abstract

**Introduction:**

Previous studies up to early 2000s found that leptospirosis incidence in humans was high across the Caribbean region (CR), yet up-to-date and reliable surveillance data are scarce. Limited research capacity in the region has further contributed to less robust characterisation of transmission drivers, perpetuating a cycle of neglect. To address these gaps and support evidence-based public health responses, this study aims to update incidence estimates in the CR by integrating data from multiple data sources, including peer-reviewed publications, surveillance reports and environmental and sociodemographic datasets.

**Methodology/Principal findings:**

We used mixed-effects hierarchical negative binomial models at the country/territory-year level, incorporating covariates (precipitation, temperature, gross domestic product, biodiversity loss, human footprint, population density, populations exposed to crop areas and frequency of extreme weather events) to estimate annual case numbers by country/territory. Temporal patterns in case fatality rate (CFR) were modelled using locally estimated scatterplot smoothing regressions. Between 2001 and 2023, we estimated 38,659 (95%CI 26,006–56,527) cases in the region. Annual incidence (cases/100,000 population) with small- to medium-sized population islands exhibiting the highest estimated incidence (Guadeloupe 23.0 (95%CI 17.2-30.3) and Saint Vincent and the Grenadines 21.3 (95%CI 11.3-36.0)). Estimated CFR increased over the study period, from 8.8% (95%CI 4.1-13.4) in 2001 to 12.2% (2.2-22.2 in 2022).

**Conclusions/Significance:**

Leptospirosis remains an important public health concern in the CR, where small island developing states bear a disproportionate burden of the disease. These findings underscore the urgent need for strengthening surveillance systems and laboratory capacity in the region, particularly in small states, to provide accurate data to prioritise public health and environmental health interventions to reduce transmission and improve diagnosis and treatment.

## Author summary

Leptospirosis is a bacterial infection that affects both animals and humans and is common across the Caribbean. Yet, many countries and territories in the region lack reliable and up-to-date information on disease incidence and mortality. This is partly due to limited laboratory capacity, inconsistent case reporting, and competing health priorities. As a result, the true burden of leptospirosis remains unclear, especially for resource-limited small island nations, which are vulnerable to potential impacts of climate change and extreme weather events on leptospirosis transmission. In this study, we combined information from peer-reviewed publications, national surveillance reports, and environmental and sociodemographic datasets to estimate annual incidence and mortality across the Caribbean. Using statistical modelling, we estimated over 38,000 cases between 2001 and 2023, with small- and medium-sized islands showing the highest incidence (cases/100,000 population). We also estimated a slight increase in case-fatality rate over time. Our findings highlight that small island developing states carry a disproportionate burden of this disease. Improving local surveillance systems and strengthening regional level collaboration are essential to help countries better identify risk, prepare for outbreaks, and reduce illness and deaths.

## Introduction

Leptospirosis is the most widespread bacterial zoonotic disease in the world [1] due to the *Leptospira*’s ability to infect a wide range of mammals and its potential to survive long periods in warm and humid environments including water and soil [2]. Globally, 1 million cases/year and nearly 60,000 deaths are estimated [3]. Across the Caribbean region (CR), previous studies up to early 2000s found that leptospirosis was highly endemic in humans, with countries like Guadeloupe, Martinique, Trinidad and Tobago, and Barbados presenting some of the highest annual reported incidence globally (ranging from 10.0 to 22.5 cases/ 100,000 population, respectively), only after Seychelles (Indian Ocean) (43.2/100,000 population) [4,5]. Additionally, between 1970 and 2012, one-third of all leptospirosis outbreaks occurred in the Latin America and Caribbean region [6], reflecting the region’s vulnerability to leptospirosis transmission [7].

Up to the early 1990s, leptospirosis was frequently associated with occupational risk and resource-poor rural environments [8–10]. More recently, outbreaks have been increasingly associated with extreme weather events, such as heavy rainfall, tropical storms and hurricanes, which are frequently followed by floods [11,12]. As global temperatures rise, these events will become more severe [13], and can further represent a heavier burden to Small Island Developing States (SIDS), which are particularly vulnerable to the impact of climate change, both economically and environmentally [13]. Additionally, SIDS are more vulnerable to biodiversity loss, as the reduction of terrestrial mammals can be associated with rodent population growth, which in turn is associated with increased leptospirosis transmission [14].

In the CR, environmental and sociodemographic conditions are ideal for *Leptospira* survival and transmission. First, the region has a very warm and humid climate, with high rainfall, particularly during the rainy season (from June to November) [1,7,15,16]. Second, rapid, unplanned urbanisation, poor sanitation in growing slums, and rising rodent population facilitate human contact with animal reservoirs and contaminated environments [12,17], driving leptospirosis outbreaks in the CR, particularly following floods. Third, traditional occupational risks remains an important source of infection, contributing to an endemic transmission associated with activities that require working in close contact with animals (e.g., dairy farmers, abattoir workers), or exposure to contaminated environments (e.g., sugarcane fieldworkers, sewerage workers) [18].

Despite its recognised burden, up-to-date and reliable leptospirosis data in the CR remain scarce [19]. Efforts to characterise leptospirosis burden face persistent challenges, frequently resulting in potential underestimation of its true incidence. Leptospirosis surveillance is inconsistent across the CR, including variation in case definitions, irregular reporting, and limited access to laboratory diagnostics to confirm cases [20]. These issues result in fragmented and outdated data, hindering effective public health planning and policy-making. The lack of robust data perpetuates a cycle of neglect, where insufficient attention and funding further limit research and surveillance. In resource-limited settings, public health systems often prioritise the most urgent health threats. In this context, shifting public health priorities can impact diagnosis and reporting capacity, particularly when multiple endemic diseases present as similar undifferentiated febrile syndromes. Recent events, such as the COVID-19 pandemic and the progressively larger dengue outbreaks in the CR, may have contributed to irregular, less robust, and poorly funded support for other endemic disease surveillance.

In the face of limited and less robust surveillance data, modelling offers a powerful tool to estimate disease burden [21], overcome data scarcity and guide decision-making. Predictive models are widely used in public health to assess epidemiological patterns and support health policy-making. In infectious disease epidemiology, statistical modelling can be used to investigate transmission dynamics, evaluate intervention impact, and identify vulnerable populations. By integrating diverse data sources, modelling can help overcome data gaps and provide actionable insights for decision-makers. To better understand the current impact of leptospirosis in the CR and support evidence-based public health responses, this study aims to provide updated incidence estimates by combining data from multiple sources, including peer-reviewed publications, surveillance reports and environmental and sociodemographic data. This aim was addressed by three objectives, (i) to estimate annual incidence by country/territory, (ii) to estimate annual mortality at the regional level, and (iii) to assess temporal trends in incidence and mortality at national and regional levels.

## Methods

### Ethics statement

All data used to build our model were obtained from publicly available sources, and no formal ethical approval was required.

### Study design

We conducted a secondary analysis of publicly available epidemiological surveillance data complemented by scientific literature focusing on island countries and territories in the CR between 2001 and 2023.

### Geographic scope

**Fig 1:**
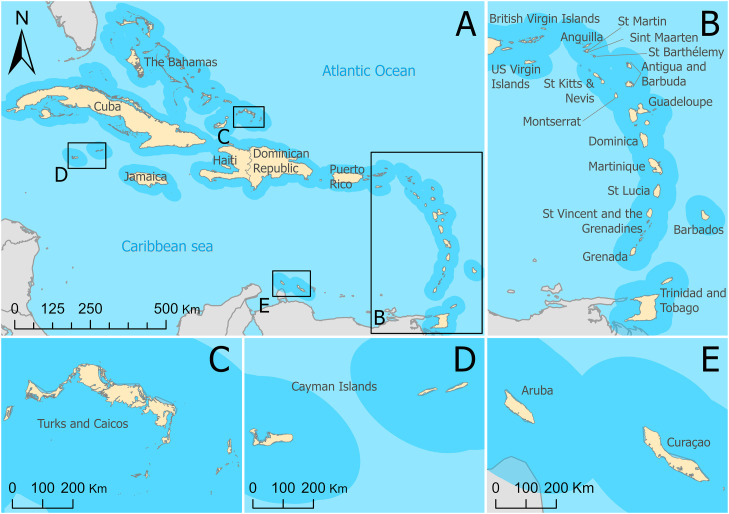
Caribbean island countries and territories included in the leptospirosis incidence model. **A)** Caribbean Region. In yellow, the 27 Caribbean island countries and territories included in our analysis. The boxes highlight: **B)** The Lesser Antilles group, and Trinidad and Tobago (top right); **C)** Turks and Caicos (bottom left), **D)** Cayman Islands (bottom middle) **E)** Aruba and Curaçao (bottom right). Base layer from: https://datacatalog.worldbank.org/search/dataset/0038272/world-bank-official-boundaries (CC BY 4.0).

In this study, we focused on 27 tropical SIDS in the CR. The particular interest in CR Islands Countries and Territories (CRICTs) was driven by higher leptospirosis transmission risk in tropical small island settings compared to continental coastal areas [3,12,14]. The CRICTs included in this study were Anguilla, Antigua and Barbuda, Aruba, Bahamas, Barbados, British Virgin Islands, Cayman Islands, Cuba, Curaçao, Dominica, Dominican Republic, Grenada, Guadeloupe, Haiti, Jamaica, Martinique, Montserrat, Puerto Rico, Saint Barthélemy, Saint Kitts and Nevis, Saint Lucia, Saint Martin, Saint Vincent and the Grenadines, Sint Maarten, Trinidad and Tobago, Turks and Caicos, and U.S. Virgin Islands (Fig 1).

### Data source and data extraction

#### Leptospirosis reported cases.

To identify all relevant data for creating our incidence estimates model, we used three main data sources. First, we incorporated the total number of reported cases by year and location extracted from peer-reviewed publications included in a scoping review of leptospirosis morbidity and mortality in the CR between 2000 and 2023 [19]. In summary, a systematic search of five databases (PubMed, Web of Science, Embase, Scopus and Latin America and Caribbean Health Literature (LILACS)) was conducted to identify publications reporting on leptospirosis epidemiology based on data collected from 2000 onwards. In each database, the search term applied was a combination of ‘Leptospirosis’ AND ‘Country/territory’, with additional publications identified though backward citation search. For the incidence model, only publications reporting incidence data were retained. For publications that reported both observed and modelled estimates of incidence/prevalence, we extracted the observed data. Further details are available in the original scoping review from which cases were extracted. The complete list of peer-reviewed publications and their case definition are available in S1 Table.

Second, we searched for grey literature on websites of health and other government departments and other relevant administrative levels to identify any epidemiological reports (e.g., weekly reports, epidemiological summaries and bulletins) communicating leptospirosis cases in a defined period. The full description of the grey literature search and included publications is available in S2 and S3 Tables.

Third, we incorporated reported cases available from the Pan American Health Organization (PAHO) Core Indicators Dashboard [22].

Case-based routine surveillance systems adopt case definition categories based on laboratory confirmation of infection. In general, confirmed cases were those with robust laboratory evidence to support leptospirosis diagnosis. Probable/suspected cases were those with insufficient or borderline laboratory evidence to support diagnoses, but highly suggestive. Non-cases are those with laboratory evidence to support an alternative diagnosis. However, nomenclature and laboratory criteria to support infection vary across countries/territories depending on available resources. In this study, the final number of cases extracted included a combination of confirmed and probable/suspected cases, incorporating the final number of cases as reported by the original data source. As the number of cases was frequently only reported as a total (combined confirmed and probable/suspected cases), this limited the possibility of extracting only laboratory confirmed cases, even when case definition was clearly provided. To prioritise laboratory confirmed cases and minimise potential duplicates, for CRICTs in which leptospirosis cases by year were reported in more than one data source, peer-reviewed studies were considered the primary source, followed by government reports. Finally, the numbers of reported cases available at the PAHO Core Indicators Dashboard were only used if no other data source was available for that CRICT and year. The final number of cases by year and CRICT used in our model can be found in S4 Table. The number of leptospirosis cases by CRICTs and year was extracted and recorded in a Microsoft Excel spreadsheet (Microsoft Corporation, Redmond, Washington, USA) [23].

#### Mortality data.

Data on mortality and case fatality rate (CFR) were obtained from two sources. First, using the same search strategy used to identify peer-reviewed publications reporting leptospirosis cases, we expanded the timeframe of the peer-reviewed studies (1979–2023) by removing any date restriction. The decision to expand the time frame was based on the limited number of peer-reviewed studies reporting mortality data identified by the scoping review. Second, we included all the epidemiological reports with mortality data, identified through grey literature search described above. Mortality data extracted from both sources were combined, following the same hierarchy previously described for cases. For each year, the number of reported deaths were combined to calculate CFR at the regional level and used to create a time trend. The number of deaths associated with leptospirosis were extracted as reported in peer-reviewed publications and grey literature, some of which combined confirmed and probable/suspected cases. The full list of peer-reviewed studies included in this step and information regarding laboratory diagnosis is provided in S5 Table.

#### Population data.

Population data by CRICTs and year were obtained through the World Bank Group [24], and data from 1995 to 2023 were extracted. Missing population data were interpolated based on a linear regression model aggregated by year and CRICT.

#### Sociodemographic and environmental variables.

Variables included in the model aimed to reflect the complex drivers of leptospirosis transmission [7] (Table 1). We extracted population density [25], number of people exposed to each land cover/land use [26], precipitation [27], temperature [28], biodiversity loss [29], human footprint [30], water flow accumulation [31], gross domestic product purchasing power parity (GDP PPP) [32–34], the annual frequency each country/territory was affected by a water related extreme weather event (heavy rainfall, storm, hurricane, flood) [35–41], the country/territory’s total area in km^2^, and annual sea surface temperature anomalies for the El Niño 3.4 region [42]. Although extremely relevant, the availability of data on animal production (livestock distribution, type and size) were limited and could not be included in our analysis. A comprehensive description of all variables extracted and tested, the source from which the data were extracted, and how the data were processed before being included in the model is available in S1 List.

**Table 1:** Variables assessed to be included in the mixed-effects model used to estimate leptospirosis incidence in the Caribbean region between 2000 and 2023, and the rationale for including each variable.

Variable	Rationale for inclusion
**Population density**	Higher population density can increase exposure risk through closer contact with contaminated environments and limited sanitation infrastructure. Population density can be used as a proxy for urbanisation [43].
**Number of people exposed to each LCLU** ^**1**^	Because the risk of leptospirosis varies across environments, and it is most relevant where people are exposed, estimating the number of people living within each LCLU class provides a more accurate measure of environment-specific transmission risk [44].
**Precipitation**	Heavy rainfall and flooding facilitate the spread of *Leptospira* bacteria by increasing waterborne transmission and environmental contamination [45,46]. It also contributes to displacement of rodent populations, increasing the geographic spread of the reservoir.
**Temperature**	Warmer temperatures can enhance bacterial survival in the environment, influencing seasonal patterns of leptospirosis outbreaks [47,48].
**Biodiversity**	Reduced biodiversity may impact host-pathogen dynamics, potentially increasing the prevalence of reservoir species like rodents [14,44].
**Human footprint**	Human activities such as urbanisation and land conversion can disrupt ecosystems and increase contact with contaminated environments [43].
**Water flow accumulation**	Areas with high water accumulation are more likely to retain contaminated water, increasing exposure risk during floods or heavy rains [49].
**GDP PPP** ^**2**^	Lower economic development is often linked to inadequate sanitation, poor housing, and limited healthcare access, all of which elevate leptospirosis risk [50].
**Extreme weather events**	The CR^3^ is frequently affected by extreme weather events, mainly heavy rainfall and hurricanes, often resulting in flooding. We extracted data (year, type of weather event, and number of events/year) from two regional climate and weather reports and from peer-reviewed publications to create a heatmap of ‘water-related’ extreme events (S1 Fig) [35–41].
**ENSO**^**4**^ **events**	Studies have documented ENSO effects on local weather driving leptospirosis outbreaks [51,52].

^1^LCLU: land cover and land use. ^2^GDP PPP: gross domestic product based on purchasing power parity. ^3^CR: Caribbean Region. ENSO: El Niño – Southern Oscillation.

In summary, for land cover/land use (LCLU), precipitation, temperature, and gross domestic product (GDP) data extracted were summarised, calculating the annual mean, between 2000 and 2023, for each CRICT. For biodiversity loss, water flow accumulation and human footprint, a unique value for each CRICT was used, and for extreme weather events, the sum of all water-related events from each year and CRICT was used. Extracted data were normalised using Z-score. Missing data were interpolated using linear models. Biodiversity loss data was unavailable for the Dutch territory of Sint Maarten, and data from the neighbouring territory of Saint Martin was used as a proxy.

### Data analysis

#### Variable selection and correlation analysis.

Univariable mixed-effects regression models were developed to examine the association between leptospirosis reported cases (outcome variable) and the socioeconomic and environmental factors (covariates) to select potential variables to be included in the multivariable model. Covariates associated with leptospirosis reported cases and with a *P*-value below 0.2 were tested for correlation to prioritise covariates to be retained in the final model. Correlation (*ρ*) between environmental and sociodemographic covariates was assessed using Pearson correlation coefficients, and pairs of covariates with a *ρ* value >±0.6 were assessed by log-marginal likelihood. Among each pair of correlated covariates, we retained the covariate in the model with the highest log-marginal likelihood**.**

#### Morbidity estimates.

**Model specification:** A multivariable, mixed-effects regression model was developed using the Integrated Nested Laplace Approximation (INLA) framework [53] to estimate leptospirosis incidence for each CRICT by year. CRICT and year were included as random effects (intercepts), modelled respectively as independent and identically distributed (iid) and as a first-order random walk (RW1) process. Penalised Complexity (PC) priors were assigned to the precision parameters of the random effects, specifying a 1% prior probability that the standard deviation of each random effect is greater than one. Sociodemographic and environmental variables were included as fixed effects. Weakly informative Gaussian priors (mean = 0, precision = 0.0001) were assigned for fixed-effect coefficients. The formal notation of the model, including model equation, link function, and specification of fixed and random effects are described in the Supporting Material.

The number of reported cases by location and year extracted from all incidence reports was used to create an overall incidence for the CR (*I*) by dividing the sum of all reported cases by the total person-years during the study period where there were cases in each country or territory. The overall incidence, *I* (0.000035), was multiplied by the population of each country/territory- year to give the expected number of cases (ϵ), assuming a homogeneous rate at the regional level. Finally, the logarithm of the expected number of cases was incorporated into the final model as an offset (log(ϵ)). By including an offset into the model, we accounted for varying population sizes (i.e., population at risk) across location-years.

**Model performance:** To identify the most suitable model for the assumed distribution, multiple model specifications were explored, including Poisson, negative binomial and zero inflated negative binomial likelihoods.

Model adequacy and potential overdispersion were evaluated through posterior predictive checks. For each fitted model, 500 posterior samples drawn from each likelihood of the fitted INLA framework, and replicated datasets were simulated. Observed and simulated summary statistics (variance-to-mean ratio and proportion of zeros) were compared, and posterior predictive *P*-values were computed to assess model fit.

Model fit was further assessed using the Deviance Information Criterion (DIC), with lower values indicating better fit. However, comparisons of DIC across models with different likelihood specifications should be interpretated with caution due to different parameterisation, including the presence of an additional dispersion parameter in the negative binomial models.

Model performance was evaluated using the root mean square error (RMSE) and cross-validation. The dataset was randomly divided into four subsets; three were used for model training and one for testing. This process was repeated 100 times, and the RMSE and the Pearson correlation between reported and estimated cases were calculated for each iteration. The mean RMSE and correlation value were reported as the final performance metric. Model selection was based on a combined evaluation of model adequacy, fit and predictive performance, with greater emphasis on posterior predictive checks when discrepancies between metrics were observed.

#### Mortality estimates.

To calculate the estimated number of deaths by CRICT and year, we used the regional case fatality rate (CFR) trend. First, the annual CFR was calculated for years in which mortality data were available. To do so, all reported deaths were divided by all reported cases (combining multiple countries when there were mortality data from more than one country/territory for that year). Annual data available were used to create a time trend plot. Then, using a Locally Estimated Scatterplot Smoothing (LOESS) model, we identified the fraction of observed data to be included in the smoothing process (span), and used these final parameters to build a LOESS model to estimate CFR with 95% CI across 2001–2023. CFR was used to estimate deaths based on the results of the incidence model (number of deaths = estimated CFR multiplied by estimated incidence). The input data are available at S6 Table.

#### Software.

R statistical programming language (R version 4.1.3, 2022-03-10) [54] was used to process spatially referenced environmental and sociodemographic data using ‘terra’ package, bivariate mixed effects models used to select variables were created suing ‘lme4’ package and statistical model to estimate incidence was fitted using ‘*INLA’* package, for data visualisation ‘*ggplot2’* was used. Google Earth Engine [27] was used to access and extract precipitation and temperature data. Esri ArcGIS PRO was used to create maps [55].

## Results

### Variable selection and model performance

Results from bivariate analysis used to select variables are summarised in S7 Table and correlation between candidate variables are presented in S2 Fig.

Comparisons between Poisson, negative binomial and zero inflated negative binomial models are presented in S8 Table.

The DIC observed for the Poisson model (373.9) was lower than for the negative binomial models (1892.6). The negative binomial model showed substantially lower RMSE (88.8) compared to the Poisson model (165.5). Posterior predictive checks indicated that both Poisson and negative binomial models overestimated variance (469.2 and 449.6, respectively) and zero counts (0.996 and 0.992, respectively), although fit improved under the negative binomial specification. Zero-inflated models did not improve model fit.

Correlation between reported and estimated cases under the final negative binomial model (Supporting Material in S1 File) was 0.813 (95%CI 0.774-0.874) (S3 Fig). Variables included in the final model and the coefficients results are presents in Table 2.

**Table 2:** Estimated coefficients and 95% credible intervals from the negative binomial mixed-effects model for leptospirosis incidence in the Caribbean region, 2001-2023.

Fixed effects	Mean (95%CI)
Intercept	-1.20 (-2.90; -0.46)
Maximum precipitation in the wettest month	0.18 (-0.01; 0.36)
Mean temperature	-0.22 (-0.64; 0.22)
GDP PPP	-0.23 (-0.53; 0.07)
Number of people exposed to crop land	0.48 (-0.20; 1.37)
Minimum variation in human footprint between 2008–2018	0.35 (-0.53; 1.33)
Biodiversity loss	-1.23 (-2.19; -0.40)
Population density	0.26 (-0.53; 1.03)
Water related extreme weather events	0.15 (-0.01; 0.32)

### Morbidity model

Between 2001 and 2023, it was estimated that 38,659 (95%CI 26,006–56,527) cases of leptospirosis occurred in the CR, with approximately 1,681 (95%CI 1,131–2,458) cases annually. Overall incidence for the study period was 4.04 (95%CI 2.72-5.90) cases/100,000 population (Table 3).

**Table 3:** Leptospirosis annual reported cases, and estimated cases, incidence and deaths for the Caribbean Region, between 2001 and 2023.

Year	Reported cases	Estimated Cases (95% CI)	Estimated incidence per 100,000 (95%CI)	Estimated Deaths^1^ (95%CI)
2001	620	1,179 (671-1,859)	3.06 (1.74-4.82)	103 (59-163)
2002	640	1,272 (776-2,017)	3.27 (1.99-5.18)	114 (69-180)
2003	781	1,401 (922-2,136)	3.57 (2.35-5.44)	127 (83-193)
2004	514	1,635 (985-2,516)	4.13 (2.49-6.35)	148 (89-228)
2005	969	1,878 (1,249-2,845)	4.71 (3.13-7.13)	170 (113-258)
2006	855	1,471 (1,047-2,091)	3.66 (2.61-5.20)	132 (94-188)
2007	1,332	2,192 (1,435-3,362)	5.41 (3.54-8.31)	196 (129-301)
2008	662	2,139 (1,280-3,403)	5.25 (3.14-8.35)	192 (115-305)
2009	325	1,659 (1,037-2,524)	4.04 (2.53-6.15)	150 (94-229)
2010	577	2,122 (1,444-3,171)	5.14 (3.49-7.67)	195 (133-292)
2011	611	2,702 (1,734-4,139)	6.50 (4.17-9.96)	252 (162-387)
2012	424	2,557 (1,690-3,836)	6.11 (4.04-9.17)	243 (160-364)
2013	667	1,862 (1,279-2,645)	4.42 (3.04-6.29)	180 (124-256)
2014	1,319	1,673 (1,223-2,358)	3.95 (2.89-5.57)	165 (120-232)
2015	880	1,311 (881-1,804)	3.08 (2.07-4.24)	132 (89-181)
2016	1,133	1,798 (1,243-2,569)	4.21 (2.91-6.01)	185 (128-264)
2017	1,273	1,859 (1,386-2,505)	4.33 (3.23-5.84)	196 (146-264)
2018	1,284	1,762 (1,303-2,394)	4.10 (3.03-5.56)	191 (141-259)
2019	1,475	1,364 (1,019-1,800)	3.15 (2.35-4.16)	152 (113-200)
2020	817	1,394 (959-1,915)	3.20 (2.20-4.39)	160 (110-219)
2021	716	1,070 (759-1,456)	2.45 (1.74-3.33)	126 (90-172)
2022	1,028	1,316 (977-1,774)	3.00 (2.23-4.05)	160 (119-216)
2023	1,139	1,043 (707-1,407)	2.37 (1.61-3.20)	NA

^1^Total number of estimated deaths per year. NA – not available.

The Dominican Republic (14,040; 8,865–21,345), Cuba (6,559; 5,0067–8,361) and Haiti (5,444; 2,407–11,556) accounted for the largest absolute number of estimated cases, representing 67.4% of all cases in the region. However, they did not rank highest in incidence rate, with estimated rates (ranks) of 3.83 (6^th^), 2.54 (14^th^), and 2.35 (15^th^) cases per 100,000 population, respectively. In contrast, smaller islands such as Guadeloupe (2,151 cases), Saint Vincent and the Grenadines (531), Martinique (1,254) and Grenada (310) contributed only to 11% of total cases but had the four highest estimated incidences: 22.99 (17.20–30.28), 21.33 (11.30–36.00), 14.14 (10.49–18.46), and 11.97 (7.45–18.44) cases per 100,000 population, respectively (Table 4).

**Table 4:** Mean annually estimated cases and incidence of leptospirosis by island countries and territories in the Caribbean Region, between 2001and 2023.

Country	Reported cases	Estimated cases (mean, [95%CI])	Estimated incidence (mean, [95%CI]/100,000 population)
Anguilla	no data	1 (0-4)	0.21 (0.00-1.22)
Antigua and Barbuda	10	40 (16-82)	2.03 (0.81-4.14)
Aruba	no data	1 (0-4)	0.04 (0.00-0.17)
Bahamas	19	22 (13-35)	0.26 (0.15-0.41)
Barbados	113	258 (161-392)	4.07 (2.54-6.19)
British Virgin Islands	no data	2 (0-11)	0.32 (0.01-1.63)
Cayman Islands	1	7 (1-22)	0.51 (0.07-1.71)
Cuba	6,589	6,559 (5,0067-8,361)	2.54 (1.94-3.24)
Curaçao	no data	1 (0-5)	0.03 (0.00-0.16)
Dominica	127	119 (87-161)	7.55 (5.51-10.22)
Dominican Republic	5,321	14,040 (8,865-21,345)	6.07 (3.83-9.23)
Grenada	114	311 (193-479)	11.97 (7.45-18.44)
Guadeloupe	1,925	2,151 (1,609-2,834)	22.99 (17.20-30.28)
Haiti	1,590	5,444 (2,407-11,556)	2.35 (1.04-4.98)
Jamaica	1,057	3,318 (2,000123-4,970)	5.23 (3.15-7.83)
Martinique	996	1,254 (930-1,638)	14.14 (10.49-18.46)
Montserrat	no data	1 (0-5)	0.93 (0.02-5.04)
Puerto Rico	1,100	2,994 (1,914-4,382)	3.65 (2.34-5.35)
Saint Barthelemy	no data	2 (0-12)	0.88 (0.01-5.50)
Sint Maarten	no data	4 (0-22)	0.49 (0.01-2.65)
St. Kitts and Nevis	23	30 (18-49)	2.83 (1.69-4.57)
St. Lucia	242	311 (218-428)	7.88 (5.53-10.84)
St. Martin	no data	24 (0-112)	3.05 (0.01-14.17)
St. Vincent and the Grenadines	89	531 (282-897)	21.33 (11.30-36.00)
Trinidad and Tobago	724	1,174 (843-1,610)	3.81 (2.74-5.23)
Turks and Caicos Islands	1	2 (0-9)	0.30 (0.04-1.13)
US Virgin Islands	no data	57 (0-346)	2.31 (0.02-13.97)

Over the 23-year study period, we did not identify any reports of leptospirosis cases in nine CRICTs (Anguilla, Aruba, British Virgin Islands, Curaçao, Montserrat, Saint Barthélemy, Sint Maarten, Saint Martin, and U.S. Virgin Islands). However, there is historical evidence of previous transmission and/or seroprevalence suggesting the occurrence of human leptospirosis in at least four of these nine CRICTs (i.e., Anguilla [56], British Virgin Islands [56], Montserrat [57], and U.S. Virgin Islands [37]), hence, our model estimated outcomes for all 27 CRICTs, even those with no reported cases. For all nine CRICTs without leptospirosis reported cases in the period investigated here, the estimated number of cases and incidence were negligible and unlikely to under or over-estimate regional numbers.

### Temporal distribution by CRICT

**Fig 2:**
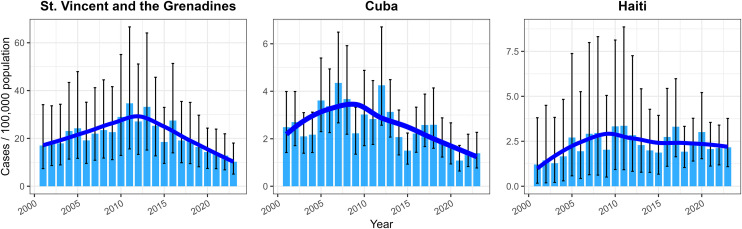
Estimated leptospirosis incidence (cases/ 100,000 population and 95% CI) by year in Saint Vincent and the Grenadines, Cuba, and Haiti, 2001-2023. The dark blue line represents an incidence time trend, using a locally estimated scatterplot smoothing regression (span 0.75).

In most CRICTs, estimated cases initially increased, peaking around 2010–2012, followed by a sustained decrease in the second half of the study period. Saint Vincent and the Grenadines and Cuba presented the greatest reduction in the total cases estimated. Comparisons between estimated cases in 2001 and 2023, show an overall reduction from 19 (8–39) to 10 (5–18) in Saint Vincent and the Grenadines and 277 (159–445) to 153 (85–251) in Cuba, with an incidence drop from 17.05 (7.38–34.13) to 10.25 (5.07–18.06) and 2.49 (1.42–3.99) to 1.39 (0.77–2.27) cases/100,000 population, respectively. Haiti was the only country where after peaking in 2011 (333 (92–878) estimated cases; 3.36 (0.92-8.86) cases/100,000 population), annual estimates plateaued (Fig 2). Graphs for all 27 CRICTs are presented in the S3 Fig.

### Mortality estimates

**Fig 3:**
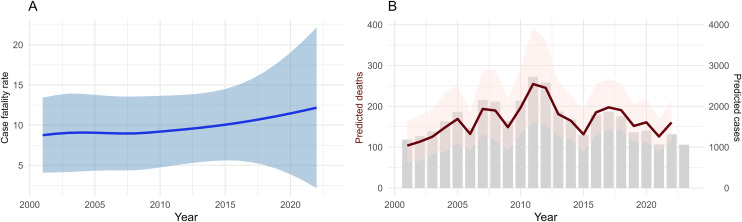
Leptospirosis estimated case fatality rate (CFR) and deaths in the Caribbean region, 2001-2023 A) Smoothed CFR over time based on the locally estimated scatterplot smoothing regression, with the light blue ribbon representing the 95% CI. **B)** Estimated deaths are shown by the red line (left y-axis), with pink ribbon representing the 95% CI. Overall Estimated cases are shown by the grey columns (right y-axis).

Data extracted from 21 publications were used to create a CFR time trend for the CR between 1968 and 2022 (S4 Fig). A LOESS curve was fitted using a span of 0.75, and the estimated CFR and 95%CI were extracted for the period between 2001–2022 (S9 Table). CFR varied from 8.8% (95% CI 4.1-13.4%) in 2001 to 12.2% (95%CI 2.2-22.2%) in 2022, suggesting an increasing trend over the study period (Fig 3a). Using the estimated CFR, mortality was estimated based on the estimated incidence (Fig 3b). Overall, 3,670 (95%CI 2,481–5,353) deaths were estimated. Peaks in mortality were mostly driven by peaks in incidence (e.g., 2011 was the year with the highest estimated incidence and mortality) (Table 1). There were insufficient data from each CRICTs to model mortality at the country or territory-level.

## Discussion

This is the first model, to our knowledge, to estimate annual leptospirosis incidence and mortality at the national level for the CR. By integrating multiple data sources and incorporating the frequency of water-related extreme weather events, our study provided up-to-date estimates of leptospirosis morbidity and mortality, identified peaks in cases associated with these events and provided a better understanding of the disease’s epidemiological patterns in the CR. Our findings underscore the continued health burden caused by leptospirosis, despite the overall decline in morbidity over the study period and the scarce literature identified by our previous scoping review [19]. Notably, our results suggest that small- to medium-population-size island countries and territories, particularly in the Lesser Antilles, exhibited disproportionately high incidence rates, frequently exceeding the regional average (4.04 cases/100,000 population). By revealing this national-level variation across the CR, our results reinforce the need to improve local surveillance and strengthen regional collaboration to support laboratory capacity and diagnosis.

The high incidence estimated for the small- to medium-population-size island countries and territories, such as Saint Vincent and the Grenadines, Guadeloupe, Grenada, and Martinique reinforces the vulnerability of smaller, resource-limited CRICTs to leptospirosis transmission [14,20]. At the same time, these CRICTs were mostly underrepresented in our scoping review [19] compared to other medium- to low-incidence CRICTs (e.g., Jamaica and Puerto Rico). This important mismatch between estimated morbidity and documentation of burden demonstrates the importance of incidence models such as ours to support public health decision-making when limited data are available. For example, in the U.S. Virgin Islands, our search did not identify any reported cases through peer-reviewed or grey literature in the last 23 years. However, the estimated incidence estimated by our model was not negligible, suggesting potential underreporting and/or environmental suitability for leptospirosis transmission. This is confirmed by a serosurvey conducted in the territory in 2019, which detected 4% seroprevalence despite no human cases documented before the Hurricanes Irma and Maria in 2017 [37]. The absence of reported cases likely reflects the neglected status of leptospirosis rather than true absence. Moreover, including CRICTs such as Anguilla, Aruba, Curaçao, and Montserrat did not inflate regional estimates and instead provided valuable insights into environmental and sociodemographic suitability for transmission, thereby supporting proactive surveillance and prevention strategies.

The Dominican Republic, Cuba, and Haiti accounted for more than two-thirds of all estimated cases, largely due to their population size. However, Guadeloupe stands out as an exception—despite its relatively small population (<500,000), it ranks among the top in both cases (2,151) and incidence (22.99/100,000 population). This aligns with previous studies documenting the historical and recent impact of leptospirosis in Guadeloupe, including outbreaks and high seroprevalence [5,58–60]. Across the study period, estimated incidence in Guadeloupe was much higher and relatively stable, in contrast to other historical high-endemicity CRICTs, such as Jamaica, Trinidad and Tobago, Cuba and Barbados, where leptospirosis cases reduced in more recent years [61], and in which our model estimated that recent incidence has been mostly below the regional average.

However, the overall decreasing trend in leptospirosis incidence across the study period should be interpreted with caution. Although it could reflect the impact of public health interventions (e.g., animal vaccination and prophylaxis for people with temporary risk [6]) and broader societal improvements (e.g., flood prevention and management), it might be biased by data scarcity, which was more pronounced in the latest years of our study. The delay between investigation and publication might explain why there were fewer studies reporting leptospirosis data in more recent years. However, data scarcity was also observed among grey literature, becoming even more challenging during and after the COVID-19 pandemic period. Additionally, the occurrence of multiple and competing health crises (e.g., Zika, dengue, COVID-19) might have impacted shifts in disease prioritisation [62,63]. These concurrent challenges may have disrupted surveillance systems, strained healthcare access, overwhelmed systems and reduced diagnostic capacity, leading to underreporting, particularly of mild cases. At the same time, severe cases (e.g., those requiring hospitalisation and deaths) are less likely to be missed by surveillance systems, which may partially explain the slight increase in CFR more recently.

This study has several limitations and data gaps. Our incidence model was based on *Leptospira*’s life cycle and human transmission framework; while we created a robust dataset integrating multiple publicly available data sources to inform drivers of transmission and reported cases, it is important to acknowledge potential impacts of limited data availability. First, case-based surveillance data for leptospirosis are frequently incomplete due to underdiagnosis, limited laboratory capacity, variability in surveillance systems, and inconsistent reporting practices across settings. Many data sources reported aggregated totals combining laboratory-confirmed and probable/suspected cases, which may have introduced misclassification bias. While inclusion of suspected cases may have led to overestimation in some settings, limited testing capacity and incomplete case ascertainment likely contributed to underreporting of true cases, particularly in resource-limited settings. Although we prioritised higher-quality sources and harmonised data where possible, these limitations may have affected burden estimates. In addition, detailed information on temporal changes in national surveillance systems and notification practices was not consistently available across countries and years. These changes may have introduced variability and discontinuities in reported incidence patterns over time. Although year was included as a temporal random effect using a first-order random walk structure to account for gradual unobserved temporal variation, abrupt changes in surveillance or reporting practices may still have influenced model outputs. Second, data on biodiversity loss were not available for the Dutch territory of Sint Maarten, requiring the use of proxy data from the neighbouring territory of Saint Martin. While this may reduce accuracy for that territory, the estimated cases were low and aligned with the absence of reported cases, suggesting minimal impact on overall model validity. Third, availability of animal data (both livestock and rodent populations) was restricted to few countries/territories, and therefore not included in the model. As humans are incidental hosts, and do not contribute to the transmission cycle, incorporating animal data would improve our model by capturing the impact of human exposure to animal reservoirs. Finally, the mortality model could not provide CRICTs-level estimates. The reported CFR varied across CRICTs and years and, unfortunately, data on healthcare access and quality were unavailable for most of the CRICTs during the study period. The lack of a reliable measure to adjust CFR based on access to healthcare hindered the possibility of providing a more comprehensive characterisation of leptospirosis mortality in the region. This study has also important strengths. We conducted a comprehensive literature review, comparing results from multiple data sources, to identify all reported cases and deaths associated with leptospirosis in the region. Additionally, by incorporating environmental and sociodemographic covariates in our estimates model, we were able to mitigate the limitations of data scarcity. Finally, we incorporated extreme water-related events to our model, allowing potential peaks in reported cases linked to those events to be addressed by the model.

Strengthening national health system data can contribute to improving data availability and reducing uncertainties regarding mortality rates across the region. Data gaps were particularly pronounced in smaller islands, which could further increase their vulnerability to leptospirosis transmission and outbreaks due lack of robust surveillance and research. As demonstrated in the U.S. Virgin Islands, the absence of reports does not equate to the absence of transmission.

Leptospirosis remains an important and often overlooked public health concern in the CR, particularly in small island countries and territories. These countries and territories often face data limitations, hindering the effort to fully characterise leptospirosis epidemiology and disease burden. This mismatch between vulnerability and data availability highlights the urgent need for more robust and coordinated regional surveillance systems. The CR could benefit from a regional integrated surveillance strategy. By adopting a unified case definition and strengthening laboratory capacity, disease monitoring can be improved by providing accurate data that can be used to guide targeted public health interventions and ensuring that leptospirosis receives appropriate attention within the regional health agenda.

## Supporting information

S1 Table Peer-reviewed publications reporting leptospirosis in the Caribbean region, between 2001 and 2023, from which leptospirosis cases were extracted. (DOCX)

S2 Table Grey literature search strategy used in Google Advanced to identify epidemiological reports of leptospirosis in the Caribbean region, between 2000 and 2023. (DOCX)

S3 Table List of documents included through grey literature search by country/territory. (DOCX)

S4 Table Reported cases by country/territory and by year extracted from peer-reviewed studies, grey literature and Pan American Health Organization (PAHO) Core Indicators Dashboard. (DOCX)

S5 Table Peer-reviewed publications used to identify the leptospirosis regional case-fatality rate. (DOCX)

S1 List A comprehensive list of variables assessed to be included in the mixed-effects model, their data sources and the spatial scale from the original data. (DOCX)

S1 Fig Extreme weather events in the Caribbean Region between 2000 and 2023, by country and territory. (DOCX)

S2 Fig Correlation Matrix showing the correlation between environmental and sociodemographic variables assessed using Pearson correlation coefficients. A ρ value ≥±0.6 indicates correlation.
(DOCX)

S6 Table Observed case fatality rate by country/territory and by year. (DOCX)

S1 File Supporting Material. Formal model specification. (DOCX)

S3 Fig Comparison between reported (observed) and predicted cases. (DOCX)

S4 Fig Observed case fatality rate between 1968 and 2022 and case fatality rate trend using Locally Estimated Scatterplot Smoothing (LOESS) model. (DOCX)

S7 Table Results from the bivariate mixed-effects regression model used to select variables to the multivariable prediction model. (DOCX)

S8 Table Comparison of model fit and predictive performance across Poisson, negative binomial and zero-inflated negative binomial models. (DOCX)

S9 Table Annual estimated cases, case fatality rate and deaths and their 95% confidence interval, between 2001 and 2022. (DOCX)
