# Supplementary material for: Estimating the burden of leptospirosis in the Caribbean: Insights from environmental and sociodemographic factors
Source: PLoS Negl Trop Dis. 2026 Jul 6;20(7):e0013876. doi: 10.1371/journal.pntd.0013876 (PMC13375137; doi:10.1371/journal.pntd.0013876)
Supplement: S1 Table — (DOCX) [file pntd.0013876.s001.docx]

| **Supporting Table 1. Peer-reviewed publications reporting leptospirosis in the Caribbean region, between 2001 and 2023, from which leptospirosis cases were extracted.** | | | |
| --- | --- | --- | --- |
| **Publication** | **Study years** | **Country** | **Case definition** |
| Golden, 2014 | 2007-2008 | Cuba | No case definition criteria were presented.  **Cases included in our analysis as reported.** |
| Herman-Storck, 2005 | 1994-2001 | Guadeloupe | Confirmed: Two laboratory criteria: 1) Enzymatic immunologic assay (EIA) IgM titre >1:400, or 2) a fourfold increase in the microscopic agglutination test (MAT) between two samples (acute and convalescent phase).  Suspected: 1) EIA IgM titre <1:400 **AND** MAT titre >1:100 (single sample available), or 2) MAT increase of less than fourfold.  Undetermined: EIA IgM <1:400 or seronegative when samples were collected too early, and MAT > 1:100 with no convalescent sera available.  **Reported cases in this study included *Confirmed, Suspected* and *Undetermined.*** |
| Herman-Storck, 2008 | 2003-2004 | Guadeloupe | Confirmed: Two laboratory criteria: 1) Enzymatic immunologic assay (EIA) IgM titre >1:400, or 2) a fourfold increase in the microscopic agglutination test (MAT) between two samples (acute and convalescent phase).  Non-case: if MAT and IgM were negative in acute and convalescent sera, or negative in a single sample collected at least 20 days after the onset of symptoms. |
| Cassadou, 2016 | 2011 | Guadeloupe and Martinique | Suspected cases were investigated with PCR, if positive, the case was confirmed. If negative, an ELISA IgM was performed; if negative (<1:400), a second sample was collected after 15 days, and another ELISA IgM was performed. If negative, it was considered a non-case. If positive (both of the first and second ELISA IgM) (>1:400), a MAT was performed for definitive confirmation. MAT were considered positive if >1:400. |
| Batchelor, 2012 | 1992-2007 | Jamaica | Confirmed: Two laboratory criteria: 1) Enzyme-Linked Immunosorbent Assay (ELISA) IgM titre >1:320, or 2) MAT > 1:200. |
| Lindo, 2013 | 2007-2008 | Jamaica | Confirmed: ELISA IgM positive (titre not provided). |
| Mohan, 2009 | 1996-2007 | Trinidad and Tobago | Confirmed: ELISA IgM > 1:640.  Undetermined: ELISA IgM >1:80 and <1:320  Non-case: ELISA IgM <1:40.  **Reported cases in this study included only *Confirmed* cases.** |
| Adesiyun, 2010 | 2006 | Trinidad and Tobago | Confirmed: ELISA IgM > 1:160 |
| Sharp, 2016 | 2010 | Puerto Rico | Confirmed: Antigen positive in a tissue specimen by immunohistochemistry (IHC), or PCR positive in tissue specimen or serum, or fourfold increase in the MAT between two paired serum samples (usually acute phase and convalescent phase), or one isolated MAT sample > 1:800.  Probable: MAT>1:100 and <1:800 in an isolated sample. |
| Jones, 2024 | 2022 | Puerto Rico | Confirmed: PCR positive  Probable: ELISA IgM positive (MAT was not possible during the study)  **Reported cases in this study included *Confirmed* and *Probable.*** |
