# Supplementary material for: Estimating the burden of leptospirosis in the Caribbean: Insights from environmental and sociodemographic factors
Source: PLoS Negl Trop Dis. 2026 Jul 6;20(7):e0013876. doi: 10.1371/journal.pntd.0013876 (PMC13375137; doi:10.1371/journal.pntd.0013876)
Supplement: S2 Table — (DOCX) [file pntd.0013876.s002.docx]

| **Supporting Table 2.** **Grey literature search strategy used in Google Advanced to identify epidemiological reports of leptospirosis in the Caribbean region, between 2000 and 2023.** | | | | |
| --- | --- | --- | --- | --- |
| **Country/territory official language** | **Search term for leptospirosis** | **Search term for cases** | **‘Country/territory name’^1^** | **‘Site domain’** |
| English | Leptospirosis | cases | Anguilla | ‘.gov.ai’ |
|  |  |  | Antigua and Barbuda | ‘.gov.ag’ |
|  |  |  | Bahamas | ‘.gov.bs’ |
|  |  |  | Barbados | ‘.gov.bb’ |
|  |  |  | British Virgin Islands | ‘.bvi.gov.vg’ |
|  |  |  | Cayman Islands | ‘.gov.ky’ |
|  |  |  | Dominica | ‘.gov.dm’ |
|  |  |  | Grenada | ‘gov.gd’ |
|  |  |  | Jamaica | ‘.gov.jm’ |
|  |  |  | Montserrat | ‘.gov.ms’ |
|  |  |  | Puerto Rico | .gov (US territory) |
|  |  |  | Saint Kitts and Nevis | ‘.gov.kn’ |
|  |  |  | Saint Lucia | ’.govt.lc’ |
|  |  |  | Sint Maarten | ‘sintmaartengov.org’ |
|  |  |  | Saint Vincent and the Grenadines | ’.gov.vc’ |
|  |  |  | Trinidad and Tobago | ‘.gov.tt’ |
|  |  |  | Turks and Caicos | ’.gov.tc’ |
|  |  |  | U.S. Virgin Islands | .gov (US territory) |
| French | Leptospirose | cas | Guadeloupe | ‘.gouv.fr’ |
|  |  |  | Haiti^2^ | ‘.gouv.ht’ |
|  |  |  | Martinique | ‘.gouv.fr’ |
|  |  |  | Saint Barthelemy | NI |
|  |  |  | Saint Martin | NI |
| Spanish | Leptospirose | casos | Cuba | ‘.gob.cu’ |
|  |  |  | Dominican Republic | ‘.gob.do’ |
|  |  |  | Puerto Rico | .gov (US territory) |
| Dutch |  |  | Aruba | ’gobierno.aw’ |
|  |  |  | Curaçao | .gobiernu.cw |
|  |  |  | Sint Maarten | ‘sintmaartengov.org’ |

1-Country/territory name was searched on the official language. 2- No search was conducted using Haitian Creole. NI- no site domain was identified.
