# Supplementary material for: Estimating the burden of leptospirosis in the Caribbean: Insights from environmental and sociodemographic factors
Source: PLoS Negl Trop Dis. 2026 Jul 6;20(7):e0013876. doi: 10.1371/journal.pntd.0013876 (PMC13375137; doi:10.1371/journal.pntd.0013876)
Supplement: S3 Table — (DOCX) [file pntd.0013876.s003.docx]

| **Supporting Table 3.** **List of documents included through grey literature search by country/territory** | | | | | | |
| --- | --- | --- | --- | --- | --- | --- |
| **Data Source** | | **Period** | **Number of cases** | **Incidence** | **Number of deaths** | **Mortality rate** |
| **Bahamas** | | | | | | |
| Ministry of Health | | 1994-2010 | Yes | No | No | No |
| Annual Report of the Chief Medical Office 2001-2003, Ministry of Health, Commonwealth of the Bahamas, 2005 | | 2001-2003 | Yes | Yes | No | No |
| **Barbados** | | | | | | |
| The Barbados Health Report, 2019 Planning and research Unit | | 2015-2018 | Yes | No | Yes | No |
| **Dominica** | | | | | | |
| Environmental Report, 2011 | | 2010 | Yes | No | No | No |
| Environmental Statistics, 2014 | | 1999-2013 | Yes | No | No | No |
| **Dominican Republic** | | | | | | |
| Casos de leptospirosis registrados por ano, segun region y provincia de residencia, 2014-2023. Oficina Nacional de Estadistica | | 2014-2023 | Yes | No | No | No |
| Infografia Enfermedades Transmissibeles | | 2019-2023 | Yes | No | No | No |
| **Guadeloupe** | | | | | | |
|  |  | 2005-2022 | Yes | Yes | Yes | Yes |
| Incidence de la leptospirose aux Antilles, 2002-2008 | | 2002-2008 | Yes | Yes | No | No |
| **Haiti** | | | | | | |
| Rapport Statistique, 2022 | |  | Yes | No | No | No |
| **Martinique** | | | | | | |
|  |  | 2005-2022 | Yes | Yes | Yes | Yes |
| Incidence de la leptospirose aux Antilles, 2002-2008 | | 2002-2008 | Yes | Yes | No | No |
| **St Lucia** | | | | | | |
| Ministry of Health, Wellness and Elderly Affairs | | 2020-2021 | Yes | No | No | No |
| **Trinidad and Tobago** | | | | | | |
| Vulnerability and Capacity Assessment (VCA) Report, January, 2019 | | 996-2007 | Yes | No | Yes | No |
