# Supplementary material for: Estimating the burden of leptospirosis in the Caribbean: Insights from environmental and sociodemographic factors
Source: PLoS Negl Trop Dis. 2026 Jul 6;20(7):e0013876. doi: 10.1371/journal.pntd.0013876 (PMC13375137; doi:10.1371/journal.pntd.0013876)
Supplement: S4 Table — (DOCX) [file pntd.0013876.s004.docx]

| **Supporting Table 4.** **Observed cases by country/territory and by year extracted from peer-reviewed studies, grey literature and Pan American Health Organization (PAHO) Core Indicators Dashboard.** | | | | | | | | | | | | | | | | | | | | | | | | |
| --- | --- | --- | --- | --- | --- | --- | --- | --- | --- | --- | --- | --- | --- | --- | --- | --- | --- | --- | --- | --- | --- | --- | --- | --- |
| **Country** | **2000** | **2001** | **2002** | **2003** | **2004** | **2005** | **2006** | **2007** | **2008** | **2009** | **2010** | **2011** | **2012** | **2013** | **2014** | **2015** | **2016** | **2017** | **2018** | **2019** | **2020** | **2021** | **2022** | **2023** |
| Anguilla |  |  |  |  |  |  |  |  |  |  |  |  |  |  | 0 | 0 | 0 | 0 | 0 | 0 | 0 | 0 | 0 | 0 |
| Antigua and Barbuda |  |  |  |  |  |  |  |  |  |  |  |  |  |  |  | 1 | 0 | 0 |  |  | 1 | 3 | 4 | 1 |
| Aruba | 0 | 0 | 0 | 0 | 0 | 0 | 0 | 0 | 0 | 0 | 0 | 0 | 0 | 0 | 0 | 0 | 0 | 0 | 0 | 0 |  |  |  |  |
| Bahamas | 0 | 0 | 1 | 0 | 0 | 1 | 0 | 3 | 4 | 0 | 1 |  | 2 | 0 | 0 | 1 | 1 | 0 | 1 | 3 | 0 | 0 | 1 | 0 |
| Barbados |  |  |  |  |  |  |  |  |  |  |  |  |  |  | 22 | 3 | 15 | 22 | 17 | 7 | 12 | 5 | 8 | 2 |
| British Virgin Islands |  |  |  |  |  |  |  |  |  |  |  |  |  |  | 0 | 0 | 0 | 0 |  |  |  | 0 | 0 |  |
| Cayman Islands |  |  |  |  |  |  |  |  |  |  |  |  |  |  | 0 | 0 | 0 | 1 |  |  |  | 0 | 0 | 0 |
| Cuba | 554 | 540 | 553 | 560 | 305 | 558 | 557 | 842 | 499 | 190 | 175 | 320 | 229 | 260 | 175 | 62 | 66 | 85 | 155 | 140 | 69 | 26 | 123 | 100 |
| Curaçao |  |  |  |  |  |  |  |  | 0 | 0 | 0 | 0 | 0 | 0 | 0 | 0 | 0 | 0 | 0 |  |  |  |  |  |
| Dominica | 0 | 0 | 2 | 2 | 4 | 6 | 4 | 2 | 2 | 1 | 11 | 29 | 5 | 8 | 10 | 10 | 16 | 7 | 7 | 0 | 1 | 0 | 0 | 0 |
| Dominican Republic |  |  |  |  |  |  |  |  |  |  |  |  |  | 139 | 533 | 466 | 778 | 792 | 580 | 569 | 210 | 288 | 263 | 703 |
| Grenada |  |  |  |  |  |  |  |  |  |  |  |  |  |  | 7 | 12 | 3 | 14 | 15 | 15 | 21 | 14 | 10 | 3 |
| Guadeloupe | 16 |  |  | 140 | 121 | 77 | 101 | 96 | 88 | 89 | 113 | 139 | 107 | 151 | 70 | 58 | 69 | 92 | 77 | 58 | 79 | 75 | 57 | 68 |
| Haiti |  |  |  |  |  |  |  |  |  |  |  |  |  |  |  | 9 |  |  | 234 | 512 | 295 | 182 | 195 | 163 |
| Jamaica | 36 | 64 | 61 | 61 | 71 | 249 | 101 | 317 |  |  |  |  |  |  |  | 133 |  |  |  |  |  |  |  |  |
| Martinique |  |  |  |  |  | 48 | 48 | 45 | 65 | 40 | 77 | 93 | 70 | 80 | 35 | 32 | 55 | 59 | 44 | 43 | 33 | 44 | 59 | 26 |
| Montserrat |  |  |  |  |  |  |  |  |  |  |  |  |  |  | 0 | 0 | 0 | 0 | 0 | 0 | 0 | 0 | 0 | 0 |
| Puerto Rico |  |  |  |  |  |  |  |  |  |  | 175 |  |  |  | 72 | 45 | 73 | 115 | 84 | 93 | 94 | 42 | 264 | 43 |
| St. Barthelemy |  |  |  |  |  |  |  |  |  |  |  |  |  |  |  |  |  |  |  |  |  |  |  |  |
| Sint Maarten |  |  |  |  |  |  |  |  |  |  |  |  |  |  |  | 0 |  | 0 | 0 |  | 0 |  |  |  |
| St. Kitts and Nevis | 1 | 0 | 0 | 3 | 1 | 2 | 0 | 4 | 0 | 1 | 8 |  |  |  | 1 |  |  | 1 | 1 | 0 | 0 | 0 | 0 | 1 |
| St. Lucia |  |  |  |  |  |  | 8 | 6 | 4 | 4 | 17 | 30 | 11 | 29 | 14 | 7 | 12 | 25 | 25 | 35 | 2 | 5 | 7 |  |
| St. Martin |  |  |  |  |  |  |  |  |  |  |  |  |  |  |  |  |  |  |  |  |  |  |  |  |
| St. Vincent and the Grenadines |  |  |  |  |  |  |  |  |  |  |  |  |  |  | 17 | 10 | 14 |  |  |  |  | 21 | 27 |  |
| Trinidad and Tobago | 19 | 16 | 23 | 15 | 12 | 28 | 36 | 17 |  |  |  |  |  |  | 363 | 30 | 32 | 58 | 44 |  |  | 11 | 10 | 29 |
| Turks and Caicos Islands |  |  |  |  |  |  |  |  |  |  |  |  |  |  | 0 | 1 | 0 | 0 | 0 | 0 | 0 | 0 | 0 | 0 |
| U.S. Virgin Islands |  |  |  |  |  |  |  |  |  |  |  |  |  |  |  |  |  |  |  |  |  |  |  |  |
