# Supplementary material for: Estimating the burden of leptospirosis in the Caribbean: Insights from environmental and sociodemographic factors
Source: PLoS Negl Trop Dis. 2026 Jul 6;20(7):e0013876. doi: 10.1371/journal.pntd.0013876 (PMC13375137; doi:10.1371/journal.pntd.0013876)
Supplement: S5 Table — (DOCX) [file pntd.0013876.s005.docx]

| **Supporting Table 5.** **Peer-reviewed publications used to identify the leptospirosis regional case-fatality rate** | | | |
| --- | --- | --- | --- |
| **Publication** | **Study years** | **Country** | **Diagnostic criteria** |
| Damude DF, Jones CJ, 1979 | 1968-1974 | Barbados | Unclear |
| Everard, 1984 | 1980-1982 | Barbados | Lab conf |
| Everard, 1995 | 1983-1991 | Barbados | Lab conf |
| Levett, 2000 | 1995 | Barbados | Lab conf |
| Cassadou, 2016 | 2011 | Guadeloupe and Martinique | Lab conf |
| Mohan, 2009 | 1996-2007 | Trinidad and Tobago | Lab conf |
| Sharp, 2016 | 2010 | Puerto Rico | Lab conf |
| Jones, 2024 | 2022 | Puerto Rico | Lab conf |
| Lhomme, 1996 | 1991 | Martinique | Lab conf |
| Chery, 2020 | 2010-2017 | St Lucia | Lab conf |
