# Supplementary material for: Estimating the burden of leptospirosis in the Caribbean: Insights from environmental and sociodemographic factors
Source: PLoS Negl Trop Dis. 2026 Jul 6;20(7):e0013876. doi: 10.1371/journal.pntd.0013876 (PMC13375137; doi:10.1371/journal.pntd.0013876)
Supplement: S1 List — (DOCX) [file pntd.0013876.s006.docx]

**Supporting List 1.** **A comprehensive list of variables assessed to be included in the mixed-effects model, their data sources and the spatial scale from the original data.**

1. **Number of people exposed to specific land use and land cover (LULC)**: Data regarding land use (e.g., agricultural, urban) and vegetation cover in a raster format with 500 metres resolution were downloaded via Google Earth Engine (GEE) (ref) from the Moderate Resolution Imaging Spectroradiometer (MODIS) Land Cover Type (MCD12Q1) Version 6.1 data product(1). We extracted annual data of LULC for all 27 island countries and territories between 2001 and 2023. The original data classifies LULC in 17 categories. In our analysis, (i) permanent wetlands, (ii) permanent snow and ice, (iii) water bodies and (iv) urban and built-up lands were kept as the original data and the remaining were grouped as (v) Forests: Evergreen Needleleaf Forests, Evergreen Broadleaf Forests, Deciduous Needleleaf Forests, Deciduous Broadleaf Forests and Mixed Forests; (vi) Savana like environment: Closed Shrublands, Open Shrublands, Woody Savannas, Savannas, Grasslands, and Barren; (vii) Croplands: Croplands and Cropland/Natural Vegetation Mosaics. The LULC maps were overlapped with population density maps, downloaded from WorldPop project (2) as raster files with 100 metres resolution for the same period. We extracted the number of people exposed to each LULC in each of the 27 countries and territories.
2. **Precipitation**: Data on precipitation were downloaded via GEE from Climate Hazards Group InfraRed Precipitation with Station data (CHIRPS) (3)in a raster format with 5 kilometres resolution, expressing millilitres per year. The original data was used to calculate the mean precipitation/year, and to extract the maximum precipitation in the wettest month per country or territory between 2001 and 2023.
3. **Temperature**: Data on temperature were downloaded via GEE from TerraClimate: Monthly Climate and Climatic Water Balance for Global Terrestrial Surfaces, University of Idaho (4) in a raster format with 1 kilometre resolution, expressing minimum, maximum and mean temperature in Celsius degree (°C) by country or territory between 2001 and 2023.
4. **Gross domestic product power purchase parity:** Data on gross domestic product power purchase parity (GDP PPP) per capita was downloaded from three sources: World Development Indicator(5)**,** Gridded global datasets for Gross Domestic Product and Human Development Index over 1990-2015(6), GDP PPP 30arcsec and the share of people living in extreme poverty (7). Each source used a different international USD year to express values. To adjust all data sources to the same currency value, we used the conversion factor available from (8). We used interpolation to estimate GDP missing values for countries and territories when possible.
5. **Water flow accumulation:** Data on water flow accumulation were downloaded from HydroShed (9) as a raster with 30-sec arc resolution (approximately 1km at the equator). Each pixel expresses the number of surrounding pixels that water would flow and accumulate. This is highly impacted by each country or territory’s area; hence, the final values extracted were weighted by the total area of each country or territory.
6. **Biodiversity loss**: Data on the percentage of native species that still remained in a country or territory was downloaded from CSIRO(10). Data was only available until 2021. We used the most updated data, 2021 as a single measure by country or territory. Data was not available for St Barthelemy. Tom completed this data we searched the closest island, with similar context (cultural, political) and size – and data from St Barthelemy was completed using data from St martin.
7. **Human footprint**: Data on human footprint (the pressure imposed on the eco-environment by changing ecological processes and natural landscapes) was downloaded from the annual records of the global Human Footprint from 2000 to 2018(11). As the whole period from 2001 and 2023 was not available, we calculated the changes in the last decade of the most update data available 2018 (2008 -2018). We extracted the mean values of changes, the maximum and the minimum. The human footprint is expressed in a scale from 0 to 50, in which 4 already shows intense human modification to the natural environment. To understand which value was more likely to impact leptospirosis transmission, we explored several cut-offs.
8. **El Niño – Southern Oscillation (ENSO) events:** Data on ENSO were obtained as monthly Niño 3.4 sea surface temperature (SST) anomalies from NOAA Climate Prediction Center (12). The Niño 3.4 index represents SST anomalies averaged over a predefined region in the equatorial Pacific Ocean and is widely used as an indicator of large-scale climatic variability. As ENSO operates at a regional scale and is not spatially resolved at the country level, the same value was assigned to all countries and territories for a given time point. Monthly values were aggregated to annual means for the period 2001 to 2023 to match the temporal resolution of the outcome data.

**References:**

1. Karra K, et al. Global land use/land cover with Sentinel-2 and deep learning. In: IGARSS 2021-2021, editor. IEEE International Geoscience and Remote Sensing Symposium.: IEEE, 2021; 2022.

2. World Bank Group. Population density (people per sq.km of land area) Washington, D.C., United States2024 [

3. Gorelick N, Hancher M, Dixon M, Ilyushchenko S, Thau D, Moore R. Google Earth Engine: Planetary-scale geospatial analysis for everyone. Remote Sensing of Environment. 2017.

4. Abatzoglou JT, Dobrowski SZ, Parks SA, Hegewisch KC. TerraClimate, a high-resolution global dataset of monthly climate and climatic water balance from 1958–2015. Scientific Data. 2018;5(1):170191.

5. World Bank Group. World Development Indicators - GDP per capita, PPP (constant 2021 international $) 2025 [Available from: [https://databank.worldbank.org/source/world-development-indicators#](https://databank.worldbank.org/source/world-development-indicators).

6. Kummu M, Taka M, Guillaume JHA. Gridded global datasets for Gross Domestic Product and Human Development Index over 1990–2015. Scientific Data. 2018;5(1):180004.

7. Our World in Data. Share of population living in extreme poverty: World Bank; 2025 [Available from: <https://ourworldindata.org/grapher/share-of-population-in-extreme-poverty>.

8. PPP conversion factor, GDP (LCU per international $) [Internet]. International Comparison Program. 2024 [cited June 16, 2025].

9. Lehner B, Verdin K, Jarvis A. New Global Hydrography Derived From Spaceborne Elevation Data. Eos, Transactions American Geophysical Union. 2008;89(10):93-4.

10. Harwood T, Ware C, Hoskins A, Ferrier S, Bush A, Golebiewski M, et al. BHI v2: Biodiversity Habitat Index: 30s global time series.: CSIRO; 2022.

11. Venter O, Sanderson EW, Magrach A, Allan JR, Beher J, Jones KR, et al. Sixteen years of change in the global terrestrial human footprint and implications for biodiversity conservation. Nature Communications. 2016;7(1):12558.

12. Rayner NA, Parker DE, Horton EB, Folland CK, Alexander LV, Rowell DP, et al. Global analyses of sea surface temperature, sea ice, and night marine air temperature since the late nineteenth century. Journal of Geophysical Research: Atmospheres. 2003;108(D14).
