# Supplementary material for: Estimating the burden of leptospirosis in the Caribbean: Insights from environmental and sociodemographic factors
Source: PLoS Negl Trop Dis. 2026 Jul 6;20(7):e0013876. doi: 10.1371/journal.pntd.0013876 (PMC13375137; doi:10.1371/journal.pntd.0013876)
Supplement: S1 Fig — (DOCX) [file pntd.0013876.s007.docx]

| **Supporting Figure 1.** Extreme weather events in the Caribbean Region between 2000 and 2023, by country and territory. | | | | | | | | | | | | | | | | | | | | | | |  |
| --- | --- | --- | --- | --- | --- | --- | --- | --- | --- | --- | --- | --- | --- | --- | --- | --- | --- | --- | --- | --- | --- | --- | --- |
|  | **Years** | | | | | | | | | | | | | | | | | | | | | | |
| **Country** | **2001** | **2002** | **2003** | **2004** | **2005** | **2006** | **2007** | **2008** | **2009** | **2010** | **2011** | **2012** | **2013** | **2014** | **2015** | **2016** | **2017** | **2018** | **2019** | **2020** | **2021** | **2022** | **2023** |
| **Anguilla** |  |  |  |  |  |  |  |  |  |  |  |  |  |  |  |  |  |  |  |  |  |  |  |
| **Antigua and Barbuda** |  |  |  |  |  |  |  |  |  |  |  |  |  |  |  |  |  |  |  |  |  |  |  |
| **Aruba** |  |  |  |  |  |  |  |  |  |  |  |  |  |  |  |  |  |  |  |  |  |  |  |
| **Bahamas** |  |  |  |  |  |  |  |  |  |  |  |  |  |  |  |  |  |  |  |  |  |  |  |
| **Barbados** |  |  |  |  |  |  | **** |  |  | ////// |  |  |  |  |  |  |  |  |  |  |  |  |  |
| **British Virgin Islands** |  |  |  |  |  |  |  |  |  |  |  |  |  |  |  |  |  |  |  |  |  |  |  |
| **Cayman Islands** |  |  |  |  |  |  |  |  |  |  |  |  |  |  |  |  |  |  |  |  |  |  |  |
| **Cuba** | ////// |  |  | ////// |  |  |  |  |  |  |  |  |  |  | ////// |  |  |  |  |  |  |  |  |
| **Curaçao** |  |  |  |  |  |  |  |  |  |  |  |  |  |  |  |  |  |  |  |  |  |  |  |
| **Dominica** |  |  |  | **** |  |  |  |  |  |  |  |  |  |  |  |  |  |  |  |  |  |  |  |
| **Dominican Republic** |  |  | **** |  | %% |  |  |  |  |  |  |  |  |  |  |  |  |  |  |  |  |  |  |
| **Grenada** |  |  |  |  |  |  |  |  |  | ////// |  |  |  |  |  |  |  |  |  |  |  |  |  |
| **Guadeloupe** |  |  |  | **** |  |  |  |  |  |  |  |  |  |  |  |  |  |  |  |  |  |  |  |
| **Haiti** |  |  | ///// |  |  |  |  |  |  | **** |  |  |  | ////// |  | ////// |  | **** |  |  | **** | **** |  |
| **Jamaica** |  |  |  |  |  |  |  |  |  |  |  |  |  | ////// |  |  |  |  |  |  |  |  |  |
| **Martinique** |  |  |  |  |  |  | **** |  |  |  |  |  |  |  |  |  |  |  |  |  |  |  |  |
| **Montserrat** |  |  |  |  |  | ### |  |  |  |  |  |  |  |  |  |  |  |  |  |  |  |  |  |
| **Puerto Rico** |  |  |  |  |  |  |  |  |  |  |  |  |  |  |  |  |  |  |  | **** |  |  |  |
| **Saint Barthelemy** |  |  |  |  |  |  |  |  |  |  |  |  |  |  |  |  |  |  |  |  |  |  |  |
| **Saint Kitts and Nevis** |  |  |  |  |  |  |  |  |  |  |  |  |  |  |  |  |  |  |  |  |  |  |  |
| **Saint Lucia** |  |  |  |  |  |  | **** |  |  | ////// |  |  |  |  |  |  |  |  |  |  |  |  |  |
| **Saint Martin** |  |  |  |  |  |  |  |  |  |  |  |  |  |  |  |  |  |  |  |  |  |  |  |
| **Saint Vincent and the Grenadines** |  |  |  |  |  |  |  |  |  | ////// |  |  |  |  |  |  |  |  |  |  | ### |  |  |
| **Sint Maarten** |  |  |  |  |  |  |  |  |  |  |  |  |  |  |  |  |  |  |  |  |  |  |  |
| **Trinidad and Tobago** |  |  |  |  |  |  |  |  |  | ////// |  |  |  |  |  |  |  |  |  |  |  |  |  |
| **Turks and Caicos** |  |  |  |  |  |  |  |  |  |  |  |  |  |  |  |  |  |  |  |  |  |  |  |
| **U.S. Virgin Islands** |  |  |  |  |  |  |  |  |  |  |  |  |  |  |  |  |  |  |  |  |  |  |  |

| **Legend:** | |
| --- | --- |
|  | Water related events (Storm, flood, hurricane, heavy rain, landslide) |
| ///// | Drought |
| **** | Earthquake |
| ### | Volcanic activity |
| %% | Wilde fire |
