# Supplementary material for: Estimating the burden of leptospirosis in the Caribbean: Insights from environmental and sociodemographic factors
Source: PLoS Negl Trop Dis. 2026 Jul 6;20(7):e0013876. doi: 10.1371/journal.pntd.0013876 (PMC13375137; doi:10.1371/journal.pntd.0013876)
Supplement: S2 Fig — A ρ value ≥±0.6 indicates correlation. (DOCX) [file pntd.0013876.s008.docx]

**Supporting Figure 2.** Correlation Matrix showing the correlation between environmental and sociodemographic variables was assessed using Pearson correlation coefficients. A *ρ* value >±0.6 indicates correlation.


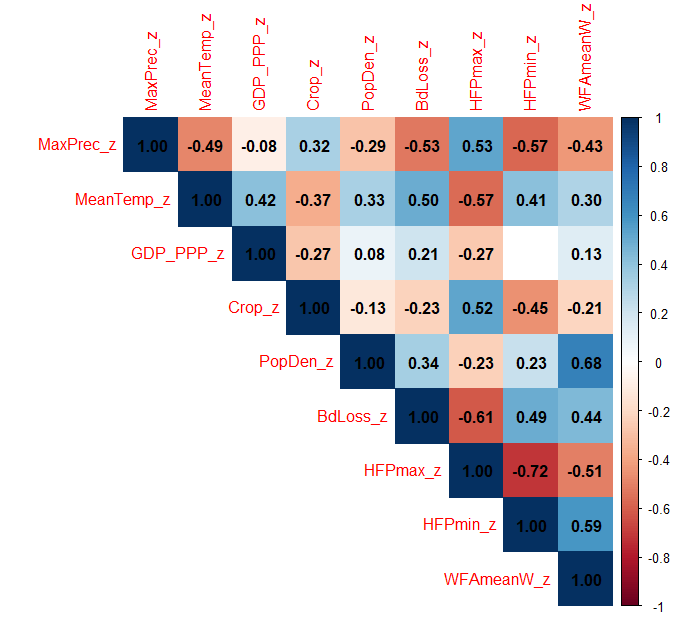


Marginal logarithm likelihood (MLL) was assessed among highly correlated pairs. Between PopDens_z (MLL= -3302.4) and WFAmeanW_z (-3305.7); BdLoss_z (-3303.4) and HFPmax_z (-3305.3); abd HFPmax_z (-3305.3) and HPFmin_z (-3306.9). As HPFmax_z was correlated with BdLoss_z and not retained in the model, HFPmin_z was retained. Extreme water related weather events was a categorical variable and was assessed individually for correlation with precipitation (0.390) and temperature (-0.460), resulting in the inclusion of it in the final model.
