# Supplementary material for: Estimating the burden of leptospirosis in the Caribbean: Insights from environmental and sociodemographic factors
Source: PLoS Negl Trop Dis. 2026 Jul 6;20(7):e0013876. doi: 10.1371/journal.pntd.0013876 (PMC13375137; doi:10.1371/journal.pntd.0013876)
Supplement: S6 Table — (DOCX) [file pntd.0013876.s009.docx]

**Supporting Table 6.** Observed case fatality rate by country/territory and by year.

|  | **Barbados** | **Dominican Republic** | **Guade-loupe** | **Martini-que** | **Puerto Rico** | **Saint Lucia** | **Trinidad & Tobago** |
| --- | --- | --- | --- | --- | --- | --- | --- |
| 1968 | 22.7 |  |  |  |  |  |  |
| 1969 | 8.1 |  |  |  |  |  |  |
| 1970 | 8.6 |  |  |  |  |  |  |
| 1971 | 48.0 |  |  |  |  |  |  |
| 1972 | 0.0 |  |  |  |  |  |  |
| 1973 | 14.3 |  |  |  |  |  |  |
| 1974 | 0.0 |  |  |  |  |  |  |
| 1980 | 11.1 |  |  |  |  |  |  |
| 1981 | 14.0 |  |  |  |  |  |  |
| 1982 | 35.1 |  |  |  |  |  |  |
| 1983 | 4.0 |  |  |  |  |  |  |
| 1984 | 0.0 |  |  |  |  |  |  |
| 1985 | 3.7 |  |  |  |  |  |  |
| 1986 | 0.0 |  |  |  |  |  |  |
| 1987 | 3.4 |  |  |  |  |  |  |
| 1988 | 3.1 |  |  |  |  |  |  |
| 1989 | 16.0 |  |  |  |  |  |  |
| 1990 | 9.5 |  |  |  | 0.0 |  |  |
| 1991 | 17.4 |  |  | 9.5 | 0.0 |  |  |
| 1992 |  |  |  |  | 0.0 |  |  |
| 1993 |  |  |  |  | 0.0 |  |  |
| 1994 |  |  |  |  | 0.0 |  |  |
| 1995 | 21.2 |  |  |  | 25.0 |  |  |
| 1996 |  |  |  |  | 16.7 |  | 13.2 |
| 1997 |  |  |  |  | 0.0 |  | 0.0 |
| 1998 |  |  |  |  | 5.9 |  | 0.0 |
| 1999 |  |  |  |  | 28.6 |  | 0.0 |
| 2000 |  |  |  |  | 8.3 |  | 15.8 |
| 2001 |  |  |  |  | 10.0 |  | 6.3 |
| 2002 |  |  |  |  | 33.3 |  | 0.0 |
| 2003 |  |  |  |  | 0.0 |  | 6.7 |
| 2004 |  |  |  |  | 3.6 |  | 16.7 |
| 2005 |  |  |  |  | 16.7 |  | 10.7 |
| 2006 |  |  |  |  | 12.5 |  | 2.8 |
| 2007 |  |  |  |  | 14.6 |  | 0.0 |
| 2008 |  |  |  |  | 6.3 |  |  |
| 2009 |  |  |  |  | 8.6 |  |  |
| 2010 |  |  |  |  | 9.1 | 5.9 |  |
| 2011 |  |  | 4.8 | 0.0 | 11.5 | 10.0 |  |
| 2012 |  |  |  |  | 8.7 | 0.0 |  |
| 2013 |  |  |  |  | 17.6 | 0.0 |  |
| 2014 |  | 10.6 |  |  | 10.3 | 0.0 |  |
| 2015 | 0.0 | 6.7 |  |  |  | 0.0 |  |
| 2016 | 6.7 | 10.3 |  |  |  | 8.3 |  |
| 2017 | 4.5 | 9.9 |  |  |  | 4.0 |  |
| 2018 | 5.9 | 9.9 |  |  |  |  |  |
| 2019 |  | 14.1 |  |  |  |  |  |
| 2020 |  | 18.1 |  |  |  |  |  |
| 2022 |  |  |  |  | 9.5 |  |  |
