# Supplementary material for: Estimating the burden of leptospirosis in the Caribbean: Insights from environmental and sociodemographic factors
Source: PLoS Negl Trop Dis. 2026 Jul 6;20(7):e0013876. doi: 10.1371/journal.pntd.0013876 (PMC13375137; doi:10.1371/journal.pntd.0013876)
Supplement: S1 File — (DOCX) [file pntd.0013876.s010.docx]

**FORMAL MODEL SPECIFICATION**

**Likelihood**

$$Y_{it}\sim\text{NegBin}(\mu_{it},\phi)$$

where $Y_{it}$ is the observed number of leptospirosis cases in country/territory $i$ ($i=1,\ldots,27$) at year $t$ ($t=2001,\ldots,2023$), and $\phi$ is the overdispersion parameter.

**Linear predictor**

$$\log(\mu_{it})=\log(E_{it})+\alpha+X_{it}^{T}\beta+u_{i}+v_{t}$$

where:

- $\log(E_{it})$ is the offset, defined as the log of expected cases under a homogeneous regional incidence rate. Specifically, $E_{it}=r\times N_{it}$, where $r=350$ cases per 100,000 population is the overall Caribbean regional incidence rate (calculated from observed cases divided by total population across all CRICT-years with available data), and $N_{it}$ is the population of CRICT $i$ at year $t$.
- $\alpha$ is the overall intercept.
- $\mathbf{X}_{it}^{T}\boldsymbol{\beta}=\sum_{j=1}^{8} \beta_{j}X_{j,it}$ represents the linear combination of $k=8$ environmental and sociodemographic fixed-effect covariates:
  - $\beta_{1}$: Maximum precipitation in the wettest month
  - $\beta_{2}$: Mean temperature
  - $\beta_{3}$: GDP PPP (gross domestic product based on purchasing power parity)
  - $\beta_{4}$: Number of people exposed to cropland
  - $\beta_{5}$: Minimum variation in human footprint
  - $\beta_{6}$: Biodiversity loss
  - $\beta_{7}$: Population density
  - $\beta_{8}$: Water-related extreme weather events (annual frequency)
- $u_{i}$ is the country/territory-specific random intercept, capturing unobserved heterogeneity in baseline leptospirosis incidence across CRICTs.
- $v_{t}$ is the year-specific random effect, capturing smooth temporal trends in regional incidence not explained by the fixed-effect covariates.

**Random effects specification**

$$u_{i}\sim\text{N}(0,\sigma_{u}^{2})\text{(iid)}$$

**Country/territory-specific random intercept**: The iid (independent and identically distributed) assumption treats each CRICT as an independent draw from a common normal distribution with mean zero and variance $\sigma_{u}^{2}$. This is an unstructured random effect with no spatial correlation imposed between neighbouring CRICTs.

$$v_{t}\sim\text{RW1}(\sigma_{v}^{2})$$

**Year-specific random effect**: A first-order random walk (RW1) prior is used to model temporal autocorrelation, allowing smooth year-to-year variation while penalizing large jumps. Under the RW1 specification:

$$v_{t}\mid v_{t-1}\sim\text{N}(v_{t-1},\sigma_{v}^{2})$$

with the constraint $\sum_{t=1}^{T} v_{t}=0$ for identifiability.

**Prior distributions**

**Precision parameters (Penalised Complexity [PC] priors):**

For the standard deviations of the random effects:

$$P(\sigma_{u}>1)=0.01,P(\sigma_{v}>1)=0.01$$

PC priors penalize complexity by favouring smaller variance components unless strongly supported by the data, with the prior probability of the standard deviation exceeding 1 set to 0.01.

**Fixed-effect coefficients (weakly informative Gaussian priors** allow the data to dominate inference while providing minimal regularization**):**

$$\beta_{j}\sim\text{N}\left( 0,\tau^{2} \right), \text{where }\tau^{2}=10,000\text{ (precision }=0.0001\text{)}$$

**Model estimation**

The model was fitted using the Integrated Nested Laplace Approximation (INLA) framework, which provides computationally efficient Bayesian inference for latent Gaussian models. Posterior marginal distributions for all parameters were obtained. Predicted incidence per 100,000 population was then obtained for each country-year. Uncertainty in predictions was quantified using 95% credible intervals derived from the posterior distributions.
