# Supplementary figures and images for: Estimating the burden of leptospirosis in the Caribbean: Insights from environmental and sociodemographic factors
Source: PLoS Negl Trop Dis. 2026 Jul 6;20(7):e0013876. doi: 10.1371/journal.pntd.0013876 (PMC13375137; doi:10.1371/journal.pntd.0013876)

**Supporting Figure 3.** Comparison between observed and predicted cases. **
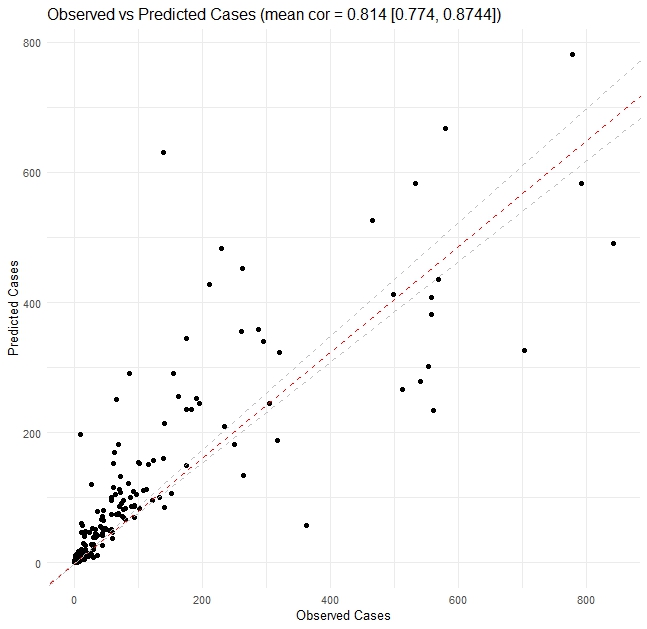
**

Supplement: S3 Fig — (DOCX) [file pntd.0013876.s011.docx]
