# Supplementary material for: Estimating the burden of leptospirosis in the Caribbean: Insights from environmental and sociodemographic factors
Source: PLoS Negl Trop Dis. 2026 Jul 6;20(7):e0013876. doi: 10.1371/journal.pntd.0013876 (PMC13375137; doi:10.1371/journal.pntd.0013876)

**Supporting Figure 4.** Observed case fatality rate between 1968 and 2022 and case fatality rate trend using Locally Estimated Scatterplot Smoothing (LOESS) model**.** In blue, span = 0.75 and in yellow, span = 1.0.
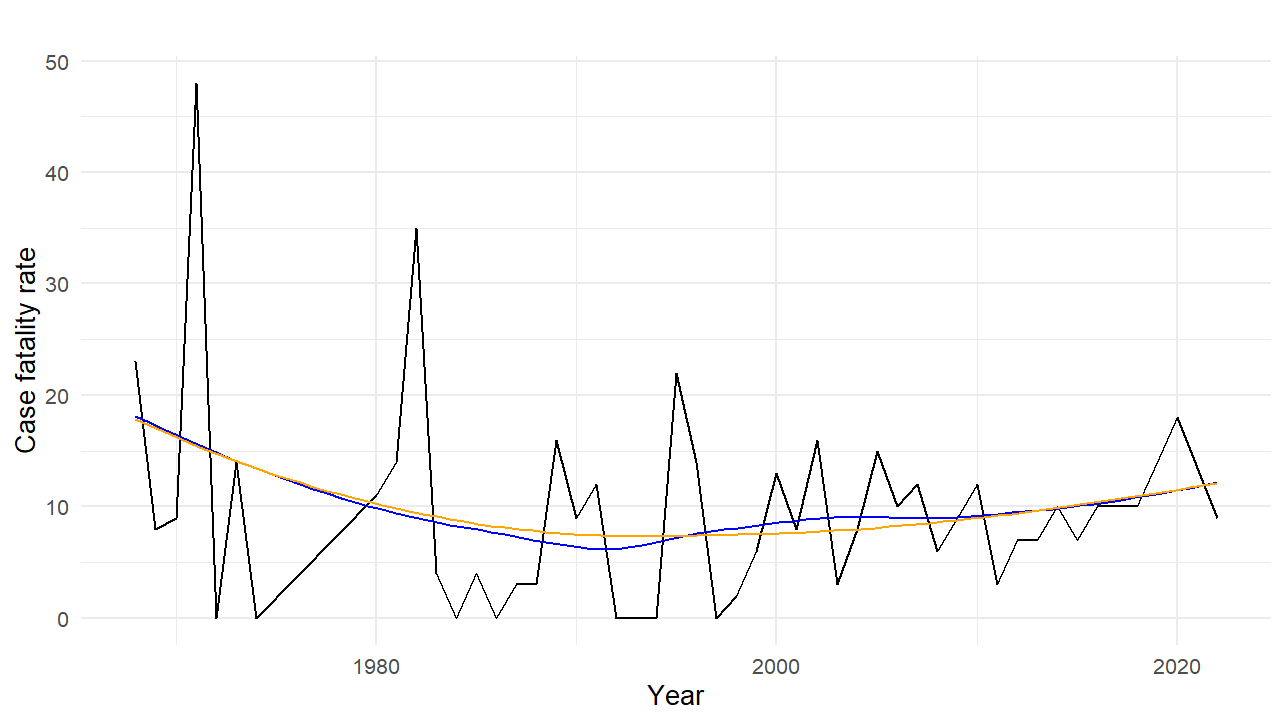

Supplement: S4 Fig — (DOCX) [file pntd.0013876.s012.docx]
