# Supplementary material for: Estimating the burden of leptospirosis in the Caribbean: Insights from environmental and sociodemographic factors
Source: PLoS Negl Trop Dis. 2026 Jul 6;20(7):e0013876. doi: 10.1371/journal.pntd.0013876 (PMC13375137; doi:10.1371/journal.pntd.0013876)
Supplement: S7 Table — (DOCX) [file pntd.0013876.s013.docx]

**Supporting Table 7. Results from the bivariate mixed-effects regression model used to select variables to the multivariable prediction model.** Variable selection criteria was a p-value <0.2 (highlighted in green).

| **Variable^1^** | **p-value**^2^ |
| --- | --- |
| Maximum precipitation in the wettest month (MaxPrec_z)^3^ | *<0.001* |
| Mean precipitation/year (MeanPrec_Z)^3^ | <0.001 |
| Mean land surface temperature/year (MeanTemp_Z) | *<0.001* |
| Annual gross domestic product power purchase parity (GDP_PPP_z) | *<0.001* |
| Number of people exposed to crop land (Crop_z) ^4^ | *<0.001* |
| Number of people exposed to forest (Forest_z) ^4^ | *<0.001* |
| Number of people exposed to savannas (Savan_z) ^4^ | *<0.001* |
| Number of people exposed to urban/built-up areas (Urban_z) ^4^ | *<0.001* |
| Population density (PopDen_z) | *<0.001* |
| Biodiversity loss (BdLoss_z) | *0.002* |
| Mean changes in human footprint between 2008-18 (HFPmean_z) | 0.486 |
| Maximum changes in human footprint between 2008-18 (HFPmax_z) | *0.017* |
| Minimum changes in human footprint between 2008-18 (HFPmin_z) | 0.116 |
| Mean weighted water flow accumulation (WFAmeanW_z) | 0.124 |
| Maximum weighted water flow accumulation (WFAmax_z) | 0.366 |
| Extreme water related weather events (water_events) | <0.001^5^ |
| Annual mean anomalies of sea surface temperature over the El Niño 3.4 region (ENSO_z) | 0.488 |

1-All variables were aggregated at the country/territory level. 2- Variables with p-value highlighted in light green were tested for correlation to be retained in the final multivariable model. 3- As maximum precipitation in the wettest month and mean precipitation both investigated the association of precipitation and leptospirosis, the variable with the highest marginal logarithm likelihood (MLL) was retained (*MaxPrec_z: MLL = -3066.6* vs MeanPrec_z: MLL = -3206.3). 4- Among all land use/land cover variables, the one with the lowest MLL was selected (Crop_z: MLL = -2974.4; Forest_z: MLL = -3011.5; Savan_z: MLL = -3293.9; Urban_z: MLL = -3241.0). 5- Extreme weather events water related was a categorical variable, the occurrence of 1, 2 and 3 events were significantly associated with leptospirosis cases.
