# Supplementary material for: Estimating the burden of leptospirosis in the Caribbean: Insights from environmental and sociodemographic factors
Source: PLoS Negl Trop Dis. 2026 Jul 6;20(7):e0013876. doi: 10.1371/journal.pntd.0013876 (PMC13375137; doi:10.1371/journal.pntd.0013876)
Supplement: S8 Table — (DOCX) [file pntd.0013876.s014.docx]

**Supporting Table 8. Comparison of model fit and predictive performance across Poisson, negative binomial and zero-inflated negative binomial models**

| **Model** | **RMSE^1^** | **DIC^2^** | **Variance to mean ratio^3^** | **Proportion of zeros^4^** |
| --- | --- | --- | --- | --- |
| Poisson | 165.5 | 373.9 | 469.2 | 0.996 |
| Negative binomial | 88.8 | 1892.6 | 449.6 | 0.992 |
| Zero inflated negative binomial | 88.5 | 1895.3 | 434.2 | 0.992 |
| *1-Root mean square error. 2- Deviance information criterion. 3- empirical variance to mean ratio was 302.6. 4- Thirty-three percent of observed counts were zeros.* | | | | |
