# Supplementary material for: Estimating the burden of leptospirosis in the Caribbean: Insights from environmental and sociodemographic factors
Source: PLoS Negl Trop Dis. 2026 Jul 6;20(7):e0013876. doi: 10.1371/journal.pntd.0013876 (PMC13375137; doi:10.1371/journal.pntd.0013876)
Supplement: S9 Table — (DOCX) [file pntd.0013876.s015.docx]

**Supporting Table 9.** Annual estimated cases, case fatality rate and deaths and their 95% confidence interval, between 2001 and 2022.

| **Year** | **Predicted cases** | | | **Case fatality rate (%)** | | | **Predicted deaths** | | |
| --- | --- | --- | --- | --- | --- | --- | --- | --- | --- |
|  | **Mean** | **2.5% CI** | **97.5% CI** | **Mean** | **2.5% CI** | **97.5% CI** | **Mean** | **2.5% CI** | **97.5% CI** |
| **2001** | 1,179 | 671 | 1,859 | 8.8 | 4.1 | 13.4 | 103 | 59 | 163 |
| **2002** | 1,272 | 776 | 2,017 | 8.9 | 4.1 | 13.7 | 114 | 69 | 180 |
| **2003** | 1,401 | 922 | 2,136 | 9.0 | 4.2 | 13.9 | 127 | 83 | 193 |
| **2004** | 1,635 | 985 | 2,516 | 9.1 | 4.2 | 13.9 | 148 | 89 | 228 |
| **2005** | 1,878 | 1,249 | 2,845 | 9.1 | 4.3 | 13.8 | 170 | 113 | 258 |
| **2006** | 1,471 | 1,047 | 2,091 | 9.0 | 4.4 | 13.6 | 132 | 94 | 188 |
| **2007** | 2,192 | 1,435 | 3,362 | 9.0 | 4.4 | 13.6 | 196 | 129 | 301 |
| **2008** | 2,139 | 1,280 | 3,403 | 9.0 | 4.4 | 13.6 | 192 | 115 | 305 |
| **2009** | 1,659 | 1,037 | 2,524 | 9.1 | 4.5 | 13.6 | 150 | 94 | 229 |
| **2010** | 2,122 | 1,444 | 3,171 | 9.2 | 4.7 | 13.7 | 195 | 133 | 292 |
| **2011** | 2,702 | 1,734 | 4,139 | 9.3 | 5.0 | 13.7 | 252 | 162 | 387 |
| **2012** | 2,557 | 1,690 | 3,836 | 9.5 | 5.2 | 13.8 | 243 | 160 | 364 |
| **2013** | 1,862 | 1,279 | 2,645 | 9.7 | 5.4 | 13.9 | 180 | 124 | 256 |
| **2014** | 1,673 | 1,223 | 2,358 | 9.8 | 5.5 | 14.2 | 165 | 120 | 232 |
| **2015** | 1,311 | 881 | 1,804 | 10.1 | 5.6 | 14.5 | 132 | 89 | 181 |
| **2016** | 1,798 | 1,243 | 2,569 | 10.3 | 5.6 | 15.0 | 185 | 128 | 264 |
| **2017** | 1,859 | 1,386 | 2,505 | 10.6 | 5.4 | 15.7 | 196 | 146 | 264 |
| **2018** | 1,762 | 1,303 | 2,394 | 10.8 | 5.0 | 16.6 | 191 | 141 | 259 |
| **2019** | 1,364 | 1,019 | 1,800 | 11.1 | 4.5 | 17.7 | 152 | 113 | 200 |
| **2020** | 1,394 | 959 | 1,915 | 11.5 | 3.9 | 19.0 | 160 | 110 | 219 |
| **2021** | 1,070 | 759 | 1,456 | 11.8 | 3.1 | 20.5 | 126 | 90 | 172 |
| **2022** | 1,316 | 977 | 1,774 | 12.2 | 2.2 | 22.2 | 160 | 119 | 216 |
| **2023** | 1,043 | 707 | 1,407 | *NA* | *NA* | *NA* | *NA* | *NA* | *NA* |
