# Supplementary material for: Estimating the burden of leptospirosis in the Caribbean: Insights from environmental and sociodemographic factors
Source: PLoS Negl Trop Dis. 2026 Jul 6;20(7):e0013876. doi: 10.1371/journal.pntd.0013876 (PMC13375137; doi:10.1371/journal.pntd.0013876)
Supplement: Attachment — Submitted filename: Response to reviewers_20260511.docx [file pntd.0013876.s017.docx]

**Additional Editor Comments:**

Firstly, apologies for the delay in the processing of this manuscript - we had some challenges recruiting reviewers.

Overall, this is an important and interesting study; however, revisions are required to improve clarity, transparency, and interpretation.

In terms of the main priorities, these should be:
1) To more clearly describe and justify the construction of the underlying dataset, including how heterogeneous data sources were combined, how case definitions were handled, and how data were harmonised across settings and time. This is key to understanding potential biases within the wide array of datasets used in the study. While much of this information is available in the supplementary materials, the key elements should be summarised in the main text, with explicit consideration of the assumptions made and the potential biases introduced.
2) The statistical modelling requires clearer reporting, including a transparent description of the model specification, presentation of all key outputs, and justification of analytical choices. In particular, the authors should more explicitly address how their methodological approach may influence the resulting burden estimates and, where possible, provide reassurance on the robustness of these findings.
3) The interpretation of results should be more cautious and better aligned with the limitations of the data and methods, particularly given the heterogeneity and uncertainty in the underlying evidence base. Some inconsistencies in reporting and presentation should also be addressed.

Please ensure that you respond to all of the specific reviewer comments in addition to the top-level summary of the main priorities given above.

R: Thank you for your careful assessment of our manuscript and for outlining these key priorities. We have revised the manuscript extensively to address each of these points.

1. *Clear description of underlying dataset*:

We have expanded the description of the construction of the underlying dataset in the Methods section (pages 6-7, lines 144-153; lines 161-177; lines 180-192), including how heterogeneous data sources were identified, combined, and harmonised across settings and years. We now explicitly describe the handling of varying case definitions and clarify the assumptions made when integrating data from peer-reviewed and grey literature. We have also added a discussion of potential biases introduced by these approaches in both the Methods and the Discussion sections.

2. *Clear reporting of the statistical modelling:*

We have revised the Methods section (pages 9-11, line 222-263) to provide a clearer and more transparent description of model specification, including model structure, assumptions and performance assessment, and variable selection procedures. Key model outputs have now been more comprehensively presented in the Results (pages 11-12 lines 287-301), and we have included additional text discussing how modelling choices may influence burden estimates, along with steps taken to assess robustness. All model outputs were updated following these revisions, resulting in corresponding updates to the tables, figures, and results presented throughout the manuscript. Due to the extent of these changes, updated numerical values within tables and figures were not individually highlighted in the marked-up version to maintain readability.

3. *Interpretation and limitations:*

We have revised the Discussion (page 18, lines 418-431) to ensure a more cautious interpretation of the findings, explicitly aligning conclusions with the limitations of the data and modelling approach. We have also addressed inconsistencies in reporting and improved clarity throughout the manuscript.

**Reviewer #1:**

**Methods**

*Objectives and Hypothesis*

The study’s objectives are exceptionally well-articulated, providing a logical roadmap for the research. Furthermore, the authors have stated a clear.

*Study Design*

The study design is robust and highly appropriate for the stated objectives. The methodology aligns perfectly with the research goals, ensuring that the data collected will provide meaningful answers to the core questions being investigated.

*Statistical Analysis*

The researchers utilized rigorous and correct statistical analyses to interpret their data. The chosen methods are well-suited for the data types, providing strong, evidence-based support for the study’s final conclusions.

*Ethical and Regulatory Compliance*

The study demonstrates a strong commitment to ethical standards.

**Results**
The manuscript is an excellently organized and technically sound piece of research. The analysis is perfectly aligned with the stated methodology, the results are presented with meticulous detail, and the supporting figures and tables are of publication-ready quality. This study provides an invaluable update on the epidemiology of leptospirosis in the Caribbean.

**Conclusions**
Does the analysis presented match the analysis plan?

Yes, the study strictly follows the methodology described in the methods section, utilizing mixed-effects hierarchical Poisson models at the country/territory-year level to estimate incidence.

The researchers correctly implemented the planned Locally Estimated Scatterplot Smoothing (LOESS) regressions to model temporal patterns in the Case Fatality Rate (CFR).

As planned, the model incorporated a wide array of pre-defined covariates, including precipitation, temperature, GDP, biodiversity loss, and extreme weather events to estimate annual case numbers.

The analysis adhered to the hierarchical data selection process, prioritizing peer-reviewed publications over government reports and PAHO data.

Are the results clearly and completely presented?

Yes, the results are presented comprehensively, featuring an estimated total of 32,311 cases in the region between 2001 and 2023.

Data is broken down clearly by year, providing observed cases, predicted cases with 95% confidence intervals, and predicted incidence rates per 100,000 people.

The study provides a thorough geographic breakdown, identifying which countries accounted for the largest absolute numbers (e.g., Dominican Republic and Cuba) versus those with the highest incidence rates (e.g., Guadeloupe).

The findings include a clear temporal analysis of the CFR, noting its increase from 8.8% in 2001 to 12.2% in 2022.

Are the figures (Tables, Images) of sufficient quality for clarity?

Yes, the figures and tables are of high quality and provide essential clarity to the data.

**Summary and General Comments**

Leptospirosis is a significant public health issue and remains largely neglected. Studies like this allow us to deepen our understanding of social determinants and their relationship with incidence in our environment. This study effectively captures its stated objective; the methods are well-detailed and precise, offering an important methodological contribution. The limitations and the treatment of the collected data are clearly outlined. The results highlight the challenges we must overcome, such as more effective prevention measures and improvements in health surveillance. Excellent work. Thank you.

R: We thank the reviewer for their time and generous evaluation of our work. We greatly appreciate the positive feedback and recognition of the importance and relevance of this study. The reviewer’s encouraging comments motivated us to further refine the manuscript to improve clarity, transparency, and robustness, and to ensure that our key massages are clearly conveyed.

**Reviewer #2:**

**Methods**

There are several aspects of the methodology requires improvement before the manuscript can be considered for publication. Specific comments:

Variable:
- Have the authors considered to include ENSO events as “regional climate” variables in model? As some studies have documented the role of ENSO on local weather and driven leptospirosis outbreaks in some Pacific islands (Rees et al., 2023 in Fiji; Weinberger et al. 2014 in New Caledonia). If it is possible, authors may try to check whether ENSO has a significant association with observed leptospirosis incidence and include this variable in the model. But, authors should check for potential collinearity.

R: We thank the reviewer for this suggestion. Following this recommendation, we explored the inclusion of ENSO as a regional climate variable in our analysis. Specifically, we extracted annual sea surface temperature anomalies for the Nino 3.4 region (ENSO index) (1) as a proxy for ENSO variability.

This variable was evaluated using the same selection criteria applied to all candidate covariates (i.e., inclusion in bivariate mixed-effects models and assessment based on statistical significance and model fit). In the bivariate analysis, ENSO was not associated with leptospirosis cases (p-value = 0.448) and was therefore not retained in the multivariable incidence estimation model.

For transparency, ENSO has been included in Table 1 (page 8, line 213), which summarises all variables assessed in the bivariate analysis, and the full results can be found in the **Supporting Table 7**.

Model:
- It would be better to separate the "Data analysis" section into sub-sections, for example: Model specification, model validation/evaluation to make it clearer.
- It would be better if authors could provide the notation of the model (Poisson mixed-effect regression model with INLA) and provide the description in detail in the Supplementary information.
- Could the authors also provide DIC or AIC, in addition to RMSE as model evaluation indices?

R: We appreciate the reviewer’s suggestion which contributed to improved clarity and transparency of the Methods sections. First, we have restructured the ‘Data analysis’ section (pages 9-11) by introducing subheadings to improve readability. We added two sub-sections under ‘Morbidity estimates’, ‘Model specification’ (line 229) and ‘Model performance’ (line 248), clearly separating the description of the modelling approach from the evaluation procedures.

Second, we included a formal notation of the model (which is now a negative-binomial mixed-effects model, after considering suggestions from reviewer #3) in the **Supporting Material (SMaterial 1)**. This included the model equation, link function, and specification of fixed and random effects.

Third, in response to the reviewer’s suggestion regarding model evaluation, we have expanded the assessment of model performance (lines 249-266). In addition to RMSE, we now report DIC to provide a more comprehensive evaluation of model fit. These results are presented under ‘Variable selection and model performance’ (page 12, lines 290-300) and in **Supporting Table 8**.

**Results**
(No Response)

**Conclusions**

(No Response)

**Summary and General Comments**

The manuscript addresses an important public health problem, leptospirosis, in the Caribbean. The study aimed to estimate the incidence and mortality rate based on routine available surveillance data, literature, and various available socio-ecological data. The study used mixed-effects hierarchical Poisson models under INLA framework. The study offers novel approach to estimate incidence and mortality rates, although some limitations exist and authors have adequately acknowledged such limitation. While the findings are interesting and substantial, yet there are several aspects that need to be considered and/or addressed before publication to improve methodological clarity and overall presentation of the paper.

R: We thank the reviewer for their time to review our manuscript and provide comments. We have revised the manuscript extensively to address the reviewer’s concern regarding the methodological aspect of the study. The changes resulted from this review were described above.

**Reviewer #3:**

**Methods**

The authors combined confirmed cases from peer-reviewed publications with reported cases derived from grey literature and international databases, assuming equivalence between these categories. However, this strategy may introduce classification bias. In the supplementary material (Table 1), different cut-off values for leptospirosis case definitions are presented. How was this potential bias addressed? The lack of distinction between confirmed and reported cases may result in systematic overestimation of disease burden. These limitations should be clearly acknowledged in the results, and the methods section should explicitly describe the categories of cases included, as well as the procedures used for data harmonization. The data were extracted from peer-reviewed publications reporting confirmed cases, as well as from grey literature and international databases with varying metadata structures and case definitions. This approach requires careful data standardization.

R: We thank the reviewer for highlighting this important concern regarding potential; classification bias arising from the combination of confirmed and reported cases across heterogeneous data sources.

We acknowledge that assuming equivalence between confirmed and probable/suspected cases my introduce misclassification and could potentially lead to overestimation of disease burden. To address this, we have revised the Methods section to more explicitly describe the categories of cases included and the procedures used for data harmonisation. Specifically, we clarified that the dataset includes a combination of laboratory-confirmed and probable/suspected cases, as reported by original sources, and that disaggregation was often not possible due to how data were reported. We also detailed the approach used to prioritise data sources and minimise duplication, with peer-reviewed studies considered the primary source, followed by government reports, and the PAHO dashboard, when multiple sources were available for the same country-year.

Pages 6-7, lines 159-174:

*“Case-based routine surveillance systems adopt case definition categories based on laboratory confirmation of infection. In general, confirmed cases were those with robust laboratory evidence to support leptospirosis diagnosis. Probable/suspected cases were those with insufficient or borderline laboratory evidence to support diagnoses, but highly suggestive. Non-cases are those with laboratory evidence to support an alternative diagnosis. However, nomenclature and laboratory criteria to support infection vary across countries/territories depending on available resources. In this study, the final number of cases extracted included a combination of confirmed and probable/suspected cases, incorporating the final number of cases as reported by the original data source. As the number of cases was frequently only reported as a total (combined confirmed and probable/suspected cases), this limited the possibility of extracting only laboratory confirmed cases, even when case definition was clearly provided. To prioritise laboratory confirmed cases and minimise potential duplicates, for CRICTs in which leptospirosis cases by year were reported in more than one data source, peer-reviewed studies were considered the primary source, followed by government reports. Finally, the numbers of reported cases available at the PAHO Core Indicators Dashboard were only used if no other data source was available for that CRICT and year. The final number of cases by year and CRICT used in our model can be found in Supporting Table 4.”*

In addition, we strengthened the discussion to explicitly acknowledge the potential for classification bias and its implications, including the possibility of overestimation of burden and increased uncertainty in the estimates.

Page 18, lines 418-426:

*“First, case-based surveillance data for leptospirosis are frequently incomplete due to underdiagnosis, limited laboratory capacity, variability in surveillance systems, and inconsistent reporting practices across settings. In addition, many data sources reported aggregated totals combining laboratory-confirmed and probable/suspected cases, which may have introduced misclassification bias. While inclusion of suspected cases may have led to overestimation in some settings, limited testing capacity and incomplete case ascertainment likely contributed to underreporting of true cases, particularly in resource-limited settings. Although we prioritised higher-quality sources and harmonised data where possible, these limitations may have affected burden estimates.”*

What were the search and data extraction criteria? Was a predefined protocol for data extraction and validation established? If so, it would be valuable to provide the registration number and make the protocol publicly available.

R: Search strategy, inclusion criteria and data extraction procedures have been previously published (2), and the reference has been included in the manuscript. To improve transparency and facilitate understanding a brief description of all procedures was added to the main text.

Page 6, lines 142-151:

*“In summary, a systematic search of five databases (PubMed, Web of Science, Embase, Scopus and Latin America and Caribbean Health Literature (LILACS)) was conducted to identified publications reporting on leptospirosis epidemiology based on data collected from 2000 onwards. In each database, the search term applied was a combination of ‘Leptospirosis’ AND ‘Country/territory’, additional publications were identified though backward citation search. For the incidence model, only publications reporting on incidence data were retained. For publications that reported both observed and modelled estimates of incidence/prevalence, we extracted the observed data. Further details are available in the original scoping review from where cases were extracted, and the complete list of peer-reviewed publications and their case definition are available in Supporting Table 1.”*

**Results**

 Given the substantial methodological limitations, interpretation of the results is premature and should be deferred until the analytical framework is revised. It is essential to first correct the methodological design, as the current results are potentially biased.

R: After reviewing the Methodology section and making the required adjustments, the Results were updated. All model outputs were updated, resulting in corresponding updates to the tables, figures, and results presented throughout the manuscript. Due to the extent of these changes, updated numerical values within tables and figures were not individually highlighted in the marked-up version to maintain readability.

**Conclusions**
Not applicable.

**Summary and General Comments**
This work is highly relevant and impactful for leptospirosis surveillance, particularly in countries (island regions) where the disease burden in the population is unclear. However, the study design and analytical choices need further description and review.

R: We thank the reviewer for their time to review our manuscript and provide comments. We have revised the manuscript extensively to address the reviewer’s concern regarding the methodological aspect of the study and updated the Results and Discussion section accordingly.

**Introduction**
Despite the strong contextual background, the introduction presents some conceptual and structural weaknesses that should be addressed. The study objective is formulated in a relatively generic manner and should be stated more clearly in the text. Based on the arguments and relevance presented, it appears that the authors aim to estimate the burden of leptospirosis across different island regions and to assess temporal trends. However, it is not evident whether the regional estimates will be aggregated or disaggregated by island and/or country.

R: We agree that the original formulation did not sufficiently specify the level of analysis or distinguish between national and regional estimates. To address this, we revised the Introduction (line 111-113) to more clearly define the scope of the study by explicitly stating the objectives underlying the main aim. We now specify that the study (i) estimates annual incidence at the country/territory level, (ii) estimates annual mortality at the regional level, and (iii) assesses temporal trends in the incidence and mortality at both national and regional scales:

*“…this study aims to provide updated incidence estimates by combining data from multiple sources, including peer-reviewed publications, surveillance reports and environmental and sociodemographic data.”). This aim was addressed by three objectives, (i) to estimate annual incidence by country/territory, (ii) to estimate annual mortality at the regional level, and (iii) to assess temporal trends in incidence and mortality at national and regional levels.”*

**Methods**
In the Geographic scope section (**line 116),** please provide the definitions of the acronyms SIDS and CR.

R: Both acronyms were previously defined. CR stands for Caribbean region and was defined in line 65. SIDS stands for Small Islands Developing States and was defined in line 76.

**Line 117** What is the surveillance period considered in this study? Is the incidence estimated on an annual basis?

R: The surveillance period considered in our study corresponds to the period between 2001 and 2023. This was added to the study design section (Lines 116 – 119):

***“Study design***

*We conducted a secondary analysis of publicly available epidemiological surveillance data complemented by scientific literature focusing on island countries and territories in the CR between 2001 and 2023.”*

The incidence was estimated by country/territory by year. This is now defined in the Introduction (lines 111-113):

“*This aim was addressed by three objectives, (i) to estimate annual incidence by country/territory, (ii) to estimate annual mortality at the regional level, and (iii) to assess temporal trends in incidence and mortality national and regional levels.”*

**Figure 1** Basic cartographic elements should be included, particularly a north arrow. It would also be helpful to add geographic boundary coordinates to facilitate spatial identification of the countries included in the study. The map legends also require adjustment, as the highlighted rectangular areas (a–d) are not described.

R: A revised version of the map was provided addressing the issues highlighted by the reviewer. The boxes in panel A highlight countries and territories with smaller territory areas that are shown in greater detail in the panels B-E. All letters are now capitalised, to facilitate the identification of the corresponding panel, and an additional description was added. We understand that readers might be less familiarised with the Caribbean region map and this correspondence might be unnoticed without further explanations. Box ‘B’ corresponds to the Lesser Antilles group shown in panel B (top right), box ‘C’ corresponds to panel C Turks and Caicos (bottom left), box ‘D’ to panel D Cayman Islands (bottom middle) and box ‘E’ to panel E Aruba and Curaçao (bottom right).

**Line 132** The data were extracted from peer-reviewed publications reporting confirmed cases, as well as from grey literature and international databases with varying metadata structures and case definitions. This approach requires careful data standardization. What were the search and data extraction criteria? Was a predefined protocol for data extraction and validation established? If so, it would be valuable to provide the registration number and make the protocol publicly available.

R: Search criteria and extraction criteria were published as a scoping review (2), referenced in the manuscript. To improve clarity and facilitate understanding, a brief explanation was added (page 6, lines 142-151)

*“In summary, a systematic search of five databases (PubMed, Web of Science, Embase, Scopus and Latin America and Caribbean Health Literature (LILACS)) was conducted to identify publications reporting on leptospirosis epidemiology based on data collected from 2000 onwards. In each database, the search term applied was a combination of ‘Leptospirosis’ AND ‘Country/territory’, with additional publications identified though backward citation search. For the incidence model, only publications reporting incidence data were retained. For publications that reported both observed and modelled estimates of incidence/prevalence, we extracted the observed data. Further details are available in the original scoping review from which cases were extracted. The complete list of peer-reviewed publications and their case definition are available in Supporting Table 1.”*

**Line 133** The study design is not clearly described, and the **criteria used to define cases** should be explicitly stated. The authors are encouraged to characterize the study as a secondary analysis of epidemiological surveillance data complemented by scientific literature.

R: We thank the reviewer for the suggestion regarding study design definition. This was incorporated to our Methods section (lines 116 – 119):

***“Study design***

*We conducted a secondary analysis of epidemiological surveillance data complemented by scientific literature focusing on island countries and territories in the CR between 2001 and 2023.”*

Regarding the criteria used to define cases, reported cases included a combination of confirmed and probable/suspected cases, varying across countries/territories. As we extracted the total number of cases as reported, we decided not to add the table with case definition by publication in the main manuscript as it could contribute to further confusion rather than clarifying our inclusion criteria. However, we agree that combining reported cases from multiple sources with different criteria can introduce potential bias and needs to be properly addressed. To acknowledge this issue and signalise this limitation to readers, we added (lines159-166):

*“Case-based routine surveillance systems adopt case definition categories based on laboratory confirmation of infection. In general, confirmed cases were those with robust laboratory evidence to support leptospirosis diagnosis. Probable/suspected cases were those with insufficient or borderline laboratory evidence to support diagnoses but highly suggestive. Non-cases are those with laboratory evidence to support an alternative diagnosis. However, nomenclature and laboratory criteria to support infection vary across countries/territories depending on available resources. In this study, the final number of cases extracted included a combination of confirmed and probable/suspected cases, incorporating the final number of cases as reported by the original data source.”*

The authors combined confirmed cases from peer-reviewed publications with “reported cases” derived from grey literature and international databases, **assuming equivalence between these categories**. However, this strategy may introduce **classification bias**. In the supplementary material (Table 1), different cut-off values for leptospirosis case definitions are presented. How was this potential bias addressed? The lack of distinction between confirmed and reported cases may result in systematic overestimation of disease burden. These limitations should be clearly acknowledged in the results, and the methods section should explicitly describe the categories of cases included, as well as the procedures used for data harmonization.

R: We agree that combining reported cases from multiple sources and using multiple criteria can introduce important bias to our prediction model. This is a common limitation in multi-country surveillance-based analyses. However, disaggregation was often not possible due to how data were reported, and even when case definition was clearly provided, cases were reported as a final number that included both laboratory confirmed and probable/suspected cases. To acknowledge potential bias, and alert readers of this limitation we added to our methods section (lines 164-174):

*“In this study, the final number of cases extracted included a combination of confirmed and probable/suspected cases, incorporating the final number of cases as reported by the original data source. As the number of cases was frequently reported as the total, this limited the possibility of extracting only laboratory confirmed cases, even when case definition was clearly provided. To prioritise laboratory confirmed cases and minimise potential duplicates, for CRICTs in which leptospirosis cases per year were reported in more than one data source, peer-reviewed studies were considered the primary source, followed by government reports. Finally, the numbers of reported cases available at the PAHO Core Indicators Dashboard were only used if no other data source was available for that CRICT and year.”*

And in the discussion. Page 18, lines 418-426:

“*First, case-based surveillance data for leptospirosis are frequently incomplete due to underdiagnosis, limited laboratory capacity, variability in surveillance systems, and inconsistent reporting practices across settings. In addition, many data sources reported aggregated totals combining laboratory-confirmed and probable/suspected cases, which may have introduced misclassification bias. While inclusion of suspected cases may have led to overestimation in some settings, limited testing capacity and incomplete case ascertainment likely contributed to underreporting of true cases, particularly in resource-limited settings. Although we prioritised higher-quality sources and harmonised data where possible, these limitations may have affected burden estimates.”*

**Line 137** The use of multiple overlapping data sources raises important concerns regarding potential duplication of records. It is unclear how the authors identified and removed duplicate cases across published studies, governmental surveillance reports, and international databases, particularly for overlapping time periods and geographic areas.

R: Potential overlap was considered. To avoid including the same case multiple time, peer-reviewed publications were considered the most reliable source, and other sources were incorporated prioritising governments reports in relation to PAHO dashboard data. This is described in lines 169-1734

*“To prioritise laboratory confirmed cases and minimise potential duplicates, for CRICTs in which leptospirosis cases by year were reported in more than one data source, peer-reviewed studies were considered the primary source, followed by government reports. Finally, the numbers of reported cases available at the PAHO Core indicators Dashboard were only used if no other data source was available for that CRICT and year.”*

**Line 156** The authors do not define the study design nor clarify whether deaths were laboratory-confirmed or merely reported. The absence of this distinction compromises the validity of the case fatality rate (CFR) estimates.

R: Similarly to data extraction of reported cases, we incorporated number of deaths as reported. Our main source of mortality data was peer-reviewed publications which all but one included only laboratory confirmed deaths. That information was added to the Supporting Table 5. Regarding grey literature, reports from two countries (Dominican Republic and Trinidad and Tobago) were not clear regarding laboratory diagnosis of reported deaths. In most countries and territories, the death of a patient reported to a surveillance system often triggers further investigation or review. For this reason, although not all deaths included were clearly stated as leptospirosis laboratory confirmed cases, we are confident that they represent confirmed cases of leptospirosis. However, we agree that this limitation should be clearly acknowledge in the manuscript, and to address this issue, we added to our Methods section (page 7, lines 186-189):

*“The number of deaths associated with leptospirosis were extracted as reported in peer-reviewed publications and grey literature, some of which combined confirmed and probable/suspected cases. The full list of peer-reviewed studies included in this step and information regarding laboratory diagnosis is provided in* ***Supporting Table 5****.”*

**Lines 157–161** Applying the same search strategy used for incidence may not be appropriate for capturing mortality data. The authors should justify the sensitivity of this approach for identifying deaths and explicitly define leptospirosis-related mortality in the text.

R: Our initial search described above aimed to include a broad spectrum of publications regarding leptospirosis epidemiology. We conducted a thorough search of incidence, prevalence, hospitalisation and mortality data associated with leptospirosis. We are confident that our search strategy, even though designed to identify leptospirosis cases, was also sensitive to identify deaths due to leptospirosis.

**Lines 158–160** How was temporal comparability ensured? The harmonization of data across incidence periods is not clearly described, particularly considering changes in notification systems and clinical management over time.

R: Temporal comparability may be affected by changes in surveillance systems, diagnostic capacity, and case definitions over time within countries. As such information was not consistently available, these changes could not be explicitly accounted for and may have introduced additional measurement variability. We have now expanded the Discussion section to acknowledge this limitation more explicitly and discuss its potential impact on burden estimates (page 18, lines 426-431):

“*In addition, detailed information on temporal changes in national surveillance systems and notification practices was not consistently available across countries and years. These changes may have introduced variability and discontinuities in reported incidence patterns over time. Although year was included as a temporal random effect using a first-order random walk structure to account for gradual unobserved temporal variation, abrupt changes in surveillance or reporting practices may still have influenced model outputs*.”

We also added the formal notation of the statistical model as part of the Supporting Material, including the temporal random effect structure (RW1), to clarify how gradual unobserved temporal variation was partially accounted for in the analysis.

**Line 165** It is unclear whether the numerator (deaths) and denominator (cases) are compatible in terms of case definition, geographic coverage, and observation period. In addition, the rationale for using the aggregated mean incidence as the primary parameter should be explained.

R: Part of this data (peer-reviewed publications) has been published and can be found in the scoping review (2) that preceded the current manuscript. It shows how data were extracted by country/territory and year to calculate case fatality rate. The same process was conducted to extract CFR from grey literature to generate our **Supporting Table 6**.

**Reviewer #4:**

**Methods**

-Are the objectives of the study clearly articulated with a clear testable hypothesis stated?
Yes.
-Is the study design appropriate to address the stated objectives?
Yes.
-Is the population clearly described and appropriate for the hypothesis being tested?
Partially. Hypothesis were not explained.

R: Thank you for this important comment. We acknowledge that not all components of this work are hypothesis-driven in the traditional sense. This is intentional and reflects the nature of the research questions and the underlying evidence gaps. While it could be possible to formulate a hypothesis such as “leptospirosis incidence is underestimated”, the study design does not directly test this proposition against a gold standard. Instead, it applies data integration and modelling approaches to generate improved estimates, which is more appropriately framed as an objective-driven and methodological contribution. No changes were made.

-Is the sample size sufficient to ensure adequate power to address the hypothesis being tested?
Yes.
-Were correct statistical analysis used to support conclusions?
Yes.
-Are there concerns about ethical or regulatory requirements being met?
Yes.

**Results**

-Does the analysis presented match the analysis plan?
Yes.
-Are the results clearly and completely presented?
No. Model results are missing.
-Are the figures (Tables, Images) of sufficient quality for clarity?
Partially. Model results are missing.

R: We thank the reviewer for highlighting the importance of sharing the regression outputs to improve transparency in our predictions. We added a table summarising the final variables included in the model and their coefficients. (Table 2, page 12, line 304).

**Conclusions**
 -Are the conclusions supported by the data presented?
Yes.
-Are the limitations of analysis clearly described?
Partially. Several limitations, regarding the relevance and interpretation of considered predictive variables were not included.

R: We thank the reviewer for their time to review our manuscript and provide comments. We have revised the manuscript extensively to clearly acknowledge the study limitations. The changes resulted from this review were described below.

-Do the authors discuss how these data can be helpful to advance our understanding of the topic under study?
Yes.
-Is public health relevance addressed?
Yes.

**Summary and General Comments**

Results

Given the substantial methodological limitations, interpretation of the results is premature and should be deferred until the analytical framework is revised. It is essential to first correct the methodological design, as the current results are potentially biased. Although the numerical findings may appear robust, they could be systematically distorted. A thorough revision of the methods and careful assessment of the identified biases are strongly recommended before drawing conclusions.

This manuscript addresses an important and policy-relevant topic: estimating the burden of leptospirosis in the Caribbean Region (CR) using hierarchical modelling approaches integrating environmental and sociodemographic covariates. The effort to compile data from multiple sources and address surveillance gaps in Small Island Developing States is commendable.
However, several conceptual clarifications and methodological transparency issues need to be addressed to strengthen the scientific robustness and interpretability of the findings. Some analytical results described in the Methods are not presented in the Results section, which limits the reader’s ability to evaluate model reliability.

R: We have revised the manuscript extensively to address each of these points.

1) Methodological limitations:
   a. We complemented the study aim with three objectives (lines 111-113), further informing readers of how data was aggregated and analysed.
   b. We included a clearer statement of case definition (lines 159-164) and acknowledge that our analysis combine laboratory confirmed cases and probable/suspected cases (lines 164-169).
   c. We also acknowledge that deaths included in our analysis might include probable/suspected cases (lines 186-189).
   d. We provided further detail regarding model specification and performance assessment (lines 229-255).
2) Results were updated based on the required changes (including Table 2 with outputs from the model in page 12, line 301). Additionally, model outputs were updated, which resulted in corresponding updates to the tables 3 and 4 (page 13, line 311 and page 14, line 329, respectively), figures 2 and 3 (page 15 line 341 and page 16, line 357), and results presented throughout the manuscript. Due to the extent of these changes, updated numerical values within tables and figures were not individually highlighted in the marked-up version to maintain readability.
3) The discussion was thoroughly reviewed to better align with methodological limitations and updated results (page 18, lines 418-431).

Detailed Comments
**Line 198.** The manuscript reports a total population of 573,031,662 for the Caribbean Region between 2001 and 2023. This figure appears unexpectedly high and the reference is not clearly provided.

R: Thank you for highlighting that this was unclear. Population data was obtained from World Bank Group (3), source with citation was provided in line 192. The number highlighted by the reviewer includes only people-years during the study period where there were leptospirosis reported cases (observed cases). We removed numbers from this section, as it was contributing to confusion and made changes to the text to reflect the correct procedures. (Page 10, line 243-245):

“*The number of reported cases by location and year extracted from all incidence reports was used to create an overall incidence for the CR (ϵ) by dividing the sum of all reported cases by the total person-years during the study period where there were cases in each country or territory.”*

**Lines 199-200**: The manuscript states that overall regional incidence was incorporated as an offset in the model. This approach is unconventional. Typically, offsets represent log(population) or exposure time. Using overall incidence as an offset may introduce circularity. Please clarify why this offset was chosen and whether log(population) was included separately, if the Poisson model was designed to include as outcome the number of cases. Also, Clarify whether overdispersion was detected and why a Poisson model was retained over a negative binomial alternative if incidence was the outcome.

R: We thank the reviewer for picking up this error on the detailed description of the model construction. The original text did not reflect this procedure and was updated to properly describe all steps:

- 1^st^ - calculating the overall incidence rate ($I$):

This was done by dividing the sum of all reported cases (20,040) by the total person-year corresponding to the study period where there were cases in each country/territory (573,031,662). The overall incidence rate was 0.000035.

- 2^nd^ – Calculating the expected number of cases (ϵ):

The overall incidence rate ($I$) was multiplied by the population of each country/territory-year.

- 3^rd^ – Calculating the offset (log(ϵ)):

The offset was obtained as log(ϵ).

The updated text now reads (page 10, lines 245-250):

***“****The overall incidence, I (0.000035), was multiplied by the population of each country/territory- year to give the expected number of cases (ϵ), assuming a homogeneous rate at the regional level. Finally, the logarithm of the expected number of cases was incorporated into the final model as an offset (log(ϵ)). By including an offset into the model, we accounted for varying population sizes (i.e., population at risk) across location-years.”*

**Line 215–216** The manuscript describes development of a multivariable Poisson mixed-effects model using INLA, including random effects and prior specifications. However, the Results section does not present the regression output. Without these results, it is difficult to evaluate the strength and direction of associations or assess biological plausibility.

R: Regression outputs from the final multivariable negative binomial model were added as a new table (Table 2, page 12, line 304), including estimated coefficients and 95% credible intervals for all covariates to improve transparency and interpretation of the modelling results.

**Line 222–224** The LOESS approach to model CFR trends is reasonable, but selection process is not fully described. Also, sensibility and specificity information from model training and testing were described in methods although results were not included in results. These metrics are essential to assess model reliability. Consider including a table summarising all covariates retained in the final model and consider making processed datasets and code publicly available to enhance reproducibility.

R: We have restructured the ‘Data analysis’ section (page 9) by introducing subheadings to improve readability. We added two sub-sections under ‘Morbidity estimates’, ‘Model specification’ (line 228) and ‘Model performance’ (line 246), clearly separating the description of the modelling approach from the evaluation procedures. The results of model performance are shown briefly under ‘Variable selection and model performance’ (Page 11, lines 287-300) and in detail in Supporting Table 8.

Additionally, a table summarising the final variables included in the model and their coefficients was included in the Results (Table 2, page 12, line 301). The final dataset used to create our model has been made available through GitHub as well as the final code used to run the analysis (<https://github.com/BeatrisMartin/Leptospirosis-Caribbean-Incidence-Modelling>).

References:

1.	Rayner NA, Parker DE, Horton EB, Folland CK, Alexander LV, Rowell DP, et al. Global analyses of sea surface temperature, sea ice, and night marine air temperature since the late nineteenth century. Journal of Geophysical Research: Atmospheres. 2003;108(D14).

2.	Martin BM, Zhang Z, Vernal S, Jian H, Nilles EJ, Furuya-Kanamori L, et al. Leptospirosis in the Caribbean Region between 2000 and 2022: A scoping review of morbidity and mortality. PLOS Neglected Tropical Diseases. 2026;20(1):e0013595.

3.	World Bank Group. World Population Prospects - Population, total. Washington, D.C., United States.2024 [Available from: <https://data.worldbank.org/indicator/SP.POP.TOTL>
