# Supplementary material for: Estimating the burden of leptospirosis in the Caribbean: Insights from environmental and sociodemographic factors
Source: PLoS Negl Trop Dis. 2026 Jul 6;20(7):e0013876. doi: 10.1371/journal.pntd.0013876 (PMC13375137; doi:10.1371/journal.pntd.0013876)
Supplement: Peer review history

# Peer review: Decision Letter 0

**DOI:** 10.1371/journal.pntd.0013876.r001

**Authors:** Max Eyre

19 Mar 2026

PNTD-D-25-02251

Estimating the burden of leptospirosis in the Caribbean: Insights from environmental and sociodemographic factors

PLOS Neglected Tropical Diseases

Dear Dr. Mario Martin,

Thank you for submitting your manuscript to PLOS Neglected Tropical Diseases. After careful consideration, we feel that it has merit but does not fully meet PLOS Neglected Tropical Diseases's publication criteria as it currently stands. Therefore, we invite you to submit a revised version of the manuscript that addresses the points raised during the review process.

Please submit your revised manuscript within by May 18 2026 11:59PM. If you will need more time than this to complete your revisions, please reply to this message or contact the journal office at plosntds@plos.org. When you're ready to submit your revision, log on to https://www.editorialmanager.com/pntd/ and select the 'Submissions Needing Revision' folder to locate your manuscript file.

Please include the following items when submitting your revised manuscript:

* A letter that responds to each point raised by the editor and reviewer(s). You should upload this letter as a separate file labeled 'Response to Reviewers'. This file does not need to include responses to any formatting updates and technical items listed in the 'Journal Requirements' section below.

* A marked-up copy of your manuscript that highlights changes made to the original version. You should upload this as a separate file labeled 'Revised Manuscript with Track Changes'.

* An unmarked version of your revised paper without tracked changes. You should upload this as a separate file labeled 'Manuscript'.

If you would like to make changes to your financial disclosure, competing interests statement, or data availability statement, please make these updates within the submission form at the time of resubmission. Guidelines for resubmitting your figure files are available below the reviewer comments at the end of this letter.

We look forward to receiving your revised manuscript.

Kind regards,

Max Thomas Eyre, PhD

Section Editor

PLOS Neglected Tropical Diseases

Max Eyre

Section Editor

PLOS Neglected Tropical Diseases

Shaden Kamhawi

co-Editor-in-Chief

PLOS Neglected Tropical Diseases

orcid.org/0000-0003-4304-636XX

Paul Brindley

co-Editor-in-Chief

PLOS Neglected Tropical Diseases

orcid.org/0000-0003-1765-0002

**Additional Editor Comments :**

Firstly, apologies for the delay in the processing of this manuscript - we had some challenges recruiting reviewers.

Overall, this is an important and interesting study; however, revisions are required to improve clarity, transparency, and interpretation.

In terms of the main priorities, these should be:

1) To more clearly describe and justify the construction of the underlying dataset, including how heterogeneous data sources were combined, how case definitions were handled, and how data were harmonised across settings and time. This is key to understanding potential biases within the wide array of datasets used in the study. While much of this information is available in the supplementary materials, the key elements should be summarised in the main text, with explicit consideration of the assumptions made and the potential biases introduced.

2) The statistical modelling requires clearer reporting, including a transparent description of the model specification, presentation of all key outputs, and justification of analytical choices. In particular, the authors should more explicitly address how their methodological approach may influence the resulting burden estimates and, where possible, provide reassurance on the robustness of these findings.

3) The interpretation of results should be more cautious and better aligned with the limitations of the data and methods, particularly given the heterogeneity and uncertainty in the underlying evidence base. Some inconsistencies in reporting and presentation should also be addressed.

Please ensure that you respond to all of the specific reviewer comments in addition to the top-level summary of the main priorities given above.

**Journal Requirements:**

1) We do not publish any copyright or trademark symbols that usually accompany proprietary names, eg ©,  ®, or TM  (e.g. next to drug or reagent names). Therefore please remove all instances of trademark/copyright symbols throughout the text, including:

- ® on page: 10.

2) Please upload all main figures as separate Figure files in .tif or .eps format. For more information about how to convert and format your figure files please see our guidelines:

https://journals.plos.org/plosntds/s/figures

3) Please amend your detailed Financial Disclosure statement. This is published with the article. It must therefore be completed in full sentences and contain the exact wording you wish to be published.

1) State what role the funders took in the study. If the funders had no role in your study, please state: "The funders had no role in study design, data collection and analysis, decision to publish, or preparation of the manuscript.".

Note: If the reviewer comments include a recommendation to cite specific previously published works, please review and evaluate these publications to determine whether they are relevant and should be cited. There is no requirement to cite these works unless the editor has indicated otherwise.

**Reviewers' Comments:**

Reviewer's Responses to Questions

**Key Review Criteria Required for Acceptance?**

As you describe the new analyses required for acceptance, please consider the following:

**Methods**

-Are the objectives of the study clearly articulated with a clear testable hypothesis stated?

-Is the study design appropriate to address the stated objectives?

-Is the population clearly described and appropriate for the hypothesis being tested?

-Is the sample size sufficient to ensure adequate power to address the hypothesis being tested?

-Were correct statistical analysis used to support conclusions?

-Are there concerns about ethical or regulatory requirements being met?

Reviewer #1: Objectives and Hypothesis

The study’s objectives are exceptionally well-articulated, providing a logical roadmap for the research. Furthermore, the authors have stated a clear.

Study Design

The study design is robust and highly appropriate for the stated objectives. The methodology aligns perfectly with the research goals, ensuring that the data collected will provide meaningful answers to the core questions being investigated.

Statistical Analysis

The researchers utilized rigorous and correct statistical analyses to interpret their data. The chosen methods are well-suited for the data types, providing strong, evidence-based support for the study’s final conclusions.

Ethical and Regulatory Compliance

The study demonstrates a strong commitment to ethical standards.

Reviewer #2: There are several aspects of the methodology requires improvement before the manuscript can be considered for publication. Specific comments:

Variable:

- Have the authors considered to include ENSO events as “regional climate” variables in model? As some studies have documented the role of ENSO on local weather and driven leptospirosis outbreaks in some Pacific islands (Rees et al., 2023 in Fiji; Weinberger et al. 2014 in New Caledonia). If it is possible, authors may try to check whether ENSO has a significant association with observed leptospirosis incidence and include this variable in the model. But, authors should check for potential collinearity.

Model:

- It would be better to separate the "Data analysis" section into sub-sections, for example: Model specification, model validation/evaluation to make it clearer.

- It would be better if authors could provide the notation of the model (Poisson mixed-effect regression model with INLA) and provide the description in detail in the Supplementary information.

- Could the authors also provide DIC or AIC, in addition to RMSE as model evaluation indices?

Reviewer #3: The authors combined confirmed cases from peer-reviewed publications with reported cases derived from grey literature and international databases, assuming equivalence between these categories. However, this strategy may introduce classification bias. In the supplementary material (Table 1), different cut-off values for leptospirosis case definitions are presented. How was this potential bias addressed? The lack of distinction between confirmed and reported cases may result in systematic overestimation of disease burden.

These limitations should be clearly acknowledged in the results, and the methods section should explicitly describe the categories of cases included, as well as the procedures used for data harmonization. he data were extracted from peer-reviewed publications reporting confirmed cases, as well as from grey literature and international databases with varying metadata structures and case definitions. This approach requires careful data standardization. What were the search and data extraction criteria? Was a predefined protocol for data extraction and validation established? If so, it would be valuable to provide the registration number and make the protocol publicly available.

Reviewer #4: -Are the objectives of the study clearly articulated with a clear testable hypothesis stated?

Yes.

-Is the study design appropriate to address the stated objectives?

Yes.

-Is the population clearly described and appropriate for the hypothesis being tested?

Partially. Hypothesis were not explained.

-Is the sample size sufficient to ensure adequate power to address the hypothesis being tested?

Yes.

-Were correct statistical analysis used to support conclusions?

Yes.

-Are there concerns about ethical or regulatory requirements being met?

Yes.

**********

**Results**

-Does the analysis presented match the analysis plan?

-Are the results clearly and completely presented?

-Are the figures (Tables, Images) of sufficient quality for clarity?

Reviewer #1: The manuscript is an excellently organized and technically sound piece of research. The analysis is perfectly aligned with the stated methodology, the results are presented with meticulous detail, and the supporting figures and tables are of publication-ready quality. This study provides an invaluable update on the epidemiology of leptospirosis in the Caribbean.

Reviewer #2: (No Response)

Reviewer #3: Given the substantial methodological limitations, interpretation of the results is premature and should be deferred until the analytical framework is revised. It is essential to first correct the methodological design, as the current results are potentially biased.

Reviewer #4: -Does the analysis presented match the analysis plan?

Yes.

-Are the results clearly and completely presented?

No. Model results are missing.

-Are the figures (Tables, Images) of sufficient quality for clarity?

Partially. Model results are missing.

**********

**Conclusions**

-Are the conclusions supported by the data presented?

-Are the limitations of analysis clearly described?

-Do the authors discuss how these data can be helpful to advance our understanding of the topic under study?

-Is public health relevance addressed?

Reviewer #1: Does the analysis presented match the analysis plan?

Yes, the study strictly follows the methodology described in the methods section, utilizing mixed-effects hierarchical Poisson models at the country/territory-year level to estimate incidence.

The researchers correctly implemented the planned Locally Estimated Scatterplot Smoothing (LOESS) regressions to model temporal patterns in the Case Fatality Rate (CFR).

As planned, the model incorporated a wide array of pre-defined covariates, including precipitation, temperature, GDP, biodiversity loss, and extreme weather events to estimate annual case numbers.

The analysis adhered to the hierarchical data selection process, prioritizing peer-reviewed publications over government reports and PAHO data.

Are the results clearly and completely presented?

Yes, the results are presented comprehensively, featuring an estimated total of 32,311 cases in the region between 2001 and 2023.

Data is broken down clearly by year, providing observed cases, predicted cases with 95% confidence intervals, and predicted incidence rates per 100,000 people.

The study provides a thorough geographic breakdown, identifying which countries accounted for the largest absolute numbers (e.g., Dominican Republic and Cuba) versus those with the highest incidence rates (e.g., Guadeloupe).

The findings include a clear temporal analysis of the CFR, noting its increase from 8.8% in 2001 to 12.2% in 2022.

Are the figures (Tables, Images) of sufficient quality for clarity?

Yes, the figures and tables are of high quality and provide essential clarity to the data.

Reviewer #2: (No Response)

Reviewer #3: Not applicable.

Reviewer #4: -Are the conclusions supported by the data presented?

Yes.

-Are the limitations of analysis clearly described?

Partially. Several limitations, regarding the relevance and interpretation of considered predictive variables were not included.

-Do the authors discuss how these data can be helpful to advance our understanding of the topic under study?

Yes.

-Is public health relevance addressed?

Yes.

**********

**Editorial and Data Presentation Modifications?**

Use this section for editorial suggestions as well as relatively minor modifications of existing data that would enhance clarity. If the only modifications needed are minor and/or editorial, you may wish to recommend “Minor Revision” or “Accept”.

Reviewer #1: (No Response)

Reviewer #2: (No Response)

Reviewer #3: Not applicable

Reviewer #4: (No Response)

**********

**Summary and General Comments**

Use this section to provide overall comments, discuss strengths/weaknesses of the study, novelty, significance, general execution and scholarship. You may also include additional comments for the author, including concerns about dual publication, research ethics, or publication ethics. If requesting major revision, please articulate the new experiments that are needed.

Reviewer #1: Leptospirosis is a significant public health issue and remains largely neglected. Studies like this allow us to deepen our understanding of social determinants and their relationship with incidence in our environment. This study effectively captures its stated objective; the methods are well-detailed and precise, offering an important methodological contribution. The limitations and the treatment of the collected data are clearly outlined. The results highlight the challenges we must overcome, such as more effective prevention measures and improvements in health surveillance. Excellent work. Thank you.

Reviewer #2: The manuscript addresses an important public health problem, leptospirosis, in the Carribean. The study aimed to estimate the incidence and mortality rate based on routine available surveillance data, literature, and various available socio-ecological data. The study used mixed-effects hierarchical Poisson models under INLA framework. The study offers novel approach to estimate incidence and mortaity rates, although some limitations exist and authors have adequately acknowledged such limitation. While the findings are interesting and substantial, yet there are several aspects that need to be considered and/or addressed before publication to improve methodological clarity and overall presentation of the paper.

Reviewer #3: This work is highly relevant and impactful for leptospirosis surveillance, particularly in countries (island regions) where the disease burden in the population is unclear. However, the study design and analytical choices need further description and review.

Introduction

Despite the strong contextual background, the introduction presents some conceptual and structural weaknesses that should be addressed. The study objective is formulated in a relatively generic manner and should be stated more clearly in the text. Based on the arguments and relevance presented, it appears that the authors aim to estimate the burden of leptospirosis across different island regions and to assess temporal trends. However, it is not evident whether the regional estimates will be aggregated or disaggregated by island and/or country.

Methods

In the Geographic scope section (line 116), please provide the definitions of the acronyms SIDS and CR.

Line 117 What is the surveillance period considered in this study? Is the incidence estimated on an annual basis?

Figure 1 Basic cartographic elements should be included, particularly a north arrow. It would also be helpful to add geographic boundary coordinates to facilitate spatial identification of the countries included in the study. The map legends also require adjustment, as the highlighted rectangular areas (a–d) are not described.

Line 132 The data were extracted from peer-reviewed publications reporting confirmed cases, as well as from grey literature and international databases with varying metadata structures and case definitions. This approach requires careful data standardization. What were the search and data extraction criteria? Was a predefined protocol for data extraction and validation established? If so, it would be valuable to provide the registration number and make the protocol publicly available.

Line 133 The study design is not clearly described, and the criteria used to define cases should be explicitly stated. The authors are encouraged to characterize the study as a secondary analysis of epidemiological surveillance data complemented by scientific literature.

The authors combined confirmed cases from peer-reviewed publications with “reported cases” derived from grey literature and international databases, assuming equivalence between these categories. However, this strategy may introduce classification bias. In the supplementary material (Table 1), different cut-off values for leptospirosis case definitions are presented. How was this potential bias addressed? The lack of distinction between confirmed and reported cases may result in systematic overestimation of disease burden.

These limitations should be clearly acknowledged in the results, and the methods section should explicitly describe the categories of cases included, as well as the procedures used for data harmonization.

Line 137 The use of multiple overlapping data sources raises important concerns regarding potential duplication of records. It is unclear how the authors identified and removed duplicate cases across published studies, governmental surveillance reports, and international databases, particularly for overlapping time periods and geographic areas.

Line 156 The authors do not define the study design nor clarify whether deaths were laboratory-confirmed or merely reported. The absence of this distinction compromises the validity of the case fatality rate (CFR) estimates.

Lines 157–161 Applying the same search strategy used for incidence may not be appropriate for capturing mortality data. The authors should justify the sensitivity of this approach for identifying deaths and explicitly define leptospirosis-related mortality in the text.

Lines 158–160 How was temporal comparability ensured? The harmonization of data across incidence periods is not clearly described, particularly considering changes in notification systems and clinical management over time.

Line 165 It is unclear whether the numerator (deaths) and denominator (cases) are compatible in terms of case definition, geographic coverage, and observation period. In addition, the rationale for using the aggregated mean incidence as the primary parameter should be explained.

Results

Given the substantial methodological limitations, interpretation of the results is premature and should be deferred until the analytical framework is revised. It is essential to first correct the methodological design, as the current results are potentially biased. Although the numerical findings may appear robust, they could be systematically distorted. A thorough revision of the methods and careful assessment of the identified biases are strongly recommended before drawing conclusions.

Reviewer #4: This manuscript addresses an important and policy-relevant topic: estimating the burden of leptospirosis in the Caribbean Region (CR) using hierarchical modelling approaches integrating environmental and sociodemographic covariates. The effort to compile data from multiple sources and address surveillance gaps in Small Island Developing States is commendable.

However, several conceptual clarifications and methodological transparency issues need to be addressed to strengthen the scientific robustness and interpretability of the findings. Some analytical results described in the Methods are not presented in the Results section, which limits the reader’s ability to evaluate model reliability.

Detailed Comments

Line 198. The manuscript reports a total population of 573,031,662 for the Caribbean Region between 2001 and 2023. This figure appears unexpectedly high and the reference is not clearly provided.

Lines 199-200: The manuscript states that overall regional incidence was incorporated as an offset in the model. This approach is unconventional. Typically, offsets represent log(population) or exposure time. Using overall incidence as an offset may introduce circularity. Please clarify why this offset was chosen and whether log(population) was included separately, if the poisson model was designed to include as outcome the number of cases. Also, Clarify whether overdispersion was detected and why a Poisson model was retained over a negative binomial alternative if incidence was the outcome.

Line 215–216 The manuscript describes development of a multivariable Poisson mixed-effects model using INLA, including random effects and prior specifications. However, the Results section does not present the regression output. Without these results, it is difficult to evaluate the strength and direction of associations or assess biological plausibility.

Line 222–224 The LOESS approach to model CFR trends is reasonable, but selection process is not fully described. Also, sensibility and especifity information from model training and testing were described in methods although results were not included in results. These metrics are essential to assess model reliability. Consider including a table summarising all covariates retained in the final model and consider making processed datasets and code publicly available to enhance reproducibility.

**********

PLOS authors have the option to publish the peer review history of their article (what does this mean?). If published, this will include your full peer review and any attached files.

If you choose “no”, your identity will remain anonymous but your review may still be made public.

**Do you want your identity to be public for this peer review?**  For information about this choice, including consent withdrawal, please see our Privacy Policy.

Reviewer #1: **Yes:** Ericka Souza Browne

Reviewer #2: No

Reviewer #3: No

Reviewer #4: No

[NOTE: If reviewer comments were submitted as an attachment file, they will be attached to this email and accessible via the submission site. Please log into your account, locate the manuscript record, and check for the action link "View Attachments". If this link does not appear, there are no attachment files.]

**Figure resubmission:**

-->While revising your submission, we strongly recommend that you use PLOS’s NAAS tool (https://ngplosjournals.pagemajik.ai/artanalysis) to test your figure files. NAAS can convert your figure files to the TIFF file type and meet basic requirements (such as print size, resolution), or provide you with a report on issues that do not meet our requirements and that NAAS cannot fix.

After uploading your figures to PLOS’s NAAS tool - https://ngplosjournals.pagemajik.ai/artanalysis, NAAS will process the files provided and display the results in the "Uploaded Files" section of the page as the processing is complete. If the uploaded figures meet our requirements (or NAAS is able to fix the files to meet our requirements), the figure will be marked as "fixed" above. If NAAS is unable to fix the files, a red "failed" label will appear above. When NAAS has confirmed that the figure files meet our requirements, please download the file via the download option, and include these NAAS processed figure files when submitting your revised manuscript.

**Reproducibility:**

To enhance the reproducibility of your results, we recommend that authors of applicable studies deposit laboratory protocols in protocols.io, where a protocol can be assigned its own identifier (DOI) such that it can be cited independently in the future. Additionally, PLOS ONE offers an option to publish peer-reviewed clinical study protocols. Read more information on sharing protocols at https://plos.org/protocols?utm_medium=editorial-email&utm_source=authorletters&utm_campaign=protocols

# Author comment: Author response to Decision Letter 1

**DOI:** 10.1371/journal.pntd.0013876.r002

13 May 2026

Attachment Submitted filename: Response to reviewers_20260511.docx

# Peer review: Decision Letter 1

**DOI:** 10.1371/journal.pntd.0013876.r003

**Authors:** Max Eyre, Max Eyre

22 Jun 2026

Dear Dr Mario Martin,

We are pleased to inform you that your manuscript 'Estimating the burden of leptospirosis in the Caribbean: Insights from environmental and sociodemographic factors' has been provisionally accepted for publication in PLOS Neglected Tropical Diseases.

Before your manuscript can be formally accepted you will need to complete some formatting changes, which you will receive in a follow up email. A member of our team will be in touch with a set of requests.

Please note that your manuscript will not be scheduled for publication until you have made the required changes, so a swift response is appreciated.

IMPORTANT: The editorial review process is now complete. PLOS will only permit corrections to spelling, formatting or significant scientific errors from this point onwards. Requests for major changes, or any which affect the scientific understanding of your work, will cause delays to the publication date of your manuscript.

Should you, your institution's press office or the journal office choose to press release your paper, you will automatically be opted out of early publication. We ask that you notify us now if you or your institution is planning to press release the article. All press must be co-ordinated with PLOS.

Thank you again for supporting Open Access publishing; we are looking forward to publishing your work in PLOS Neglected Tropical Diseases.

Best regards,

Max Thomas Eyre, PhD

Section Editor

PLOS Neglected Tropical Diseases

Max Eyre

Section Editor

PLOS Neglected Tropical Diseases

Shaden Kamhawi

co-Editor-in-Chief

PLOS Neglected Tropical Diseases

orcid.org/0000-0003-4304-636XX

Paul Brindley

co-Editor-in-Chief

PLOS Neglected Tropical Diseases

orcid.org/0000-0003-1765-0002

***********************************************************

p.p1 {margin: 0.0px 0.0px 0.0px 0.0px; line-height: 16.0px; font: 14.0px Arial; color: #323333; -webkit-text-stroke: #323333}span.s1 {font-kerning: none

Reviewer's Responses to Questions

**Key Review Criteria Required for Acceptance?**

As you describe the new analyses required for acceptance, please consider the following:

**Methods**

-Are the objectives of the study clearly articulated with a clear testable hypothesis stated?

-Is the study design appropriate to address the stated objectives?

-Is the population clearly described and appropriate for the hypothesis being tested?

-Is the sample size sufficient to ensure adequate power to address the hypothesis being tested?

-Were correct statistical analysis used to support conclusions?

-Are there concerns about ethical or regulatory requirements being met?

Reviewer #1: Minor Revision

Justification regarding the El Niño Variable (ENSO): In the document's track changes/history, Reviewer 2 suggested including ENSO. You tested this variable in the bivariate analysis, found it was not significant (p = 0.448), and moved it to Supplementary Table 7.

Tip: Ensure that in the final text of the Methods section (around lines 200–213), it is explicitly clear in a short sentence that "the ENSO index was evaluated but not retained in the multivariable model due to lack of statistical significance", preventing future reviewers from raising the same question.

Transparency in Misclassification Bias: Reviewer 3 expressed strong concern regarding the mixing of "confirmed cases" and "probable/suspected cases" extracted from gray literature. Although you harmonized this very well by adding justifications to the methodology (lines 159–166) and the limitations section in the Discussion, make sure that Table 1 (which summarizes the variables) or the results table titles consistently use the term "reported cases" instead of just "incidence" or "confirmed cases", thereby mitigating allegations of overestimating the disease burden.

**********

**Results**

-Does the analysis presented match the analysis plan?

-Are the results clearly and completely presented?

-Are the figures (Tables, Images) of sufficient quality for clarity?

Reviewer #1: Yes, the results are presented in a clear and comprehensive manner, with significant improvements in raw data transparency.

Transparency in Estimates: The results not only provide the crude rates but also break down the annual incidence estimates per 100,000 inhabitants by specific sub-regions and territories in the Caribbean, along with their respective confidence intervals (or credibility/prediction intervals).

Presentation of Coefficients: The inclusion of Table 2 directly within the main text—detailing the final variables included in the model and their respective coefficients and incidence rate ratios (IRR)—has resolved a common omission in modeling manuscripts. This allows the reader to see the actual weight of factors such as precipitation and human footprint.

Comprehensive Supplementary Materials: Extensive tables detailing model performance and goodness-of-fit metrics (such as AIC/BIC or prediction errors) have been allocated to the appendices to avoid overwhelming the main text, maintaining a fluid reading experience without compromising scientific completeness.

**********

**Conclusions**

-Are the conclusions supported by the data presented?

-Are the limitations of analysis clearly described?

-Do the authors discuss how these data can be helpful to advance our understanding of the topic under study?

-Is public health relevance addressed?

Reviewer #1: Yes, the conclusions are supported by the data and have been properly calibrated following the rounds of review.

Alignment with the Results: The authors conclude that leptospirosis is endemic across much of the Caribbean and strongly influenced by environmental factors (such as precipitation) and socio-environmental factors (such as the human footprint). This directly reflects the statistically significant incidence rate ratios (IRRs) presented in Table 2 of the Results.

Moderation in Tone (Important Adjustment): In previous versions, there was a risk of overestimating the actual disease burden due to the inclusion of heterogeneous surveillance data. In response to the reviewers' comments (especially Reviewer 3), the authors adjusted the tone of the conclusions. Instead of declaring the modeled estimates as absolute truths of epidemiological incidence, the text now correctly frames them as "estimates of the probable burden under current data limitations." This demonstrates scientific rigor and avoids extrapolations unsupported by the model.

**********

**Editorial and Data Presentation Modifications?**

Use this section for editorial suggestions as well as relatively minor modifications of existing data that would enhance clarity. If the only modifications needed are minor and/or editorial, you may wish to recommend “Minor Revision” or “Accept”.

Reviewer #1: Based on a thorough review of the revised manuscript (PNTD-D-25-02251R1) and the authors' responses to the previous round of reviews, the paper is in a very strong position. The authors have successfully addressed the major methodological concerns, particularly regarding model validation (implementing the leave-one-year-out cross-validation) and transparency in handling non-significant variables (such as ENSO).

To fully meet the strict publication standards of PLOS Neglected Tropical Diseases, the following minor editorial, stylistic, and data presentation modifications are suggested to enhance the manuscript's overall clarity and precision.

1. Abstract and Author Summary (Minor Refinements)Grammar/Word Choice Clarification: In the Conclusions/Significance section of the Abstract, the sentence currently reads:"...to reduce transmission and improve diagnose and treatment."

Modification: Change "diagnose" (verb) to "diagnosis" (noun) to match "treatment." The corrected phrase should read: "...to reduce transmission and improve diagnosis and treatment."

Author summary accessibility: The Author Summary is well-written, but ensure that any mention of the modeling framework avoids excessive statistical jargon. Explaining that the model "accounts for differences in reporting across different island territories by grouping data hierarchically" helps a non-specialist reader understand the "hierarchical" nature of your negative binomial model without needing a background in statistics.

2. Consistency in Data TerminologyAdopting "Reported Cases" over "Incidence": As highlighted during the review process, the input data relies heavily on a mix of confirmed and suspected cases from heterogeneous surveillance systems and grey literature.

Modification: Throughout the Results section text and in the column headers of Table 1, ensure consistency by using terms like "Reported Incidence" or "Recorded Cases" rather than a generic or absolute "Incidence" or "True Burden". This minor textual adjustment continuously reinforces the appropriate boundary of your data to the reader.

3. Textual transitions for excluded variables explicitly addressing excluded predictors: The authors did excellent work moving non-significant variables (like El Niño Southern Oscillation - ENSO and sea surface temperature anomalies) to Supporting Tables 7 and 8 after the bivariate analysis.

Modification: In the Results section text (under the "Variable selection and model performance" subsection), add a brief, single-sentence explicit transition.

For example:"Variables that did not show a statistically significant association during the bivariate screening, such as the ENSO index (p = 0.448), were excluded from the final multivariable framework (see Supporting Table 7 for full bivariate outputs)." This prevents future readers or editors from wondering why macro-climatic factors like El Niño were omitted from the final presentation.

**********

**Summary and General Comments**

Use this section to provide overall comments, discuss strengths/weaknesses of the study, novelty, significance, general execution and scholarship. You may also include additional comments for the author, including concerns about dual publication, research ethics, or publication ethics. If requesting major revision, please articulate the new experiments that are needed.

Reviewer #1: (No Response)

**********

PLOS authors have the option to publish the peer review history of their article (what does this mean?). If published, this will include your full peer review and any attached files.

If you choose “no”, your identity will remain anonymous but your review may still be made public.

**Do you want your identity to be public for this peer review?**  For information about this choice, including consent withdrawal, please see our Privacy Policy.

Reviewer #1: **Yes:** Ericka Souza Browne

# Editor report: Acceptance letter

**DOI:** 10.1371/journal.pntd.0013876.r004

**Authors:** Max Eyre, Max Eyre

Dear Dr Mario Martin,

We are delighted to inform you that your manuscript, "Estimating the burden of leptospirosis in the Caribbean: Insights from environmental and sociodemographic factors," has been formally accepted for publication in PLOS Neglected Tropical Diseases.

We have now passed your article onto the PLOS Production Department who will complete the rest of the publication process. All authors will receive a confirmation email upon publication.

The corresponding author will soon be receiving a typeset proof for review, to ensure errors have not been introduced during production. Please review the PDF proof of your manuscript carefully, as this is the last chance to correct any scientific or type-setting errors. Please note that major changes, or those which affect the scientific understanding of the work, will likely cause delays to the publication date of your manuscript. Note: Proofs for Front Matter articles (Editorial, Viewpoint, Symposium, Review, etc...) are generated on a different schedule and may not be made available as quickly.

Soon after your final files are uploaded, the early version of your manuscript will be published online unless you opted out of this process. The date of the early version will be your article's publication date. The final article will be published to the same URL, and all versions of the paper will be accessible to readers.

For Research Articles, you will receive an invoice from PLOS for your publication fee after your manuscript has reached the completed accept phase. If you receive an email requesting payment before acceptance or for any other service, this may be a phishing scheme. Learn how to identify phishing emails and protect your accounts at https://explore.plos.org/phishing.

Thank you again for supporting open-access publishing; we are looking forward to publishing your work in PLOS Neglected Tropical Diseases.

Best regards,

Shaden Kamhawi

co-Editor-in-Chief

PLOS Neglected Tropical Diseases

Paul Brindley

co-Editor-in-Chief

PLOS Neglected Tropical Diseases
